# Discovery of DNA Topoisomerase I Inhibitors with Low-Cytotoxicity Based on Virtual Screening from Natural Products

**DOI:** 10.3390/md15070217

**Published:** 2017-07-09

**Authors:** Lan-Ting Xin, Lu Liu, Chang-Lun Shao, Ri-Lei Yu, Fang-Ling Chen, Shi-Jun Yue, Mei Wang, Zhong-Long Guo, Ya-Chu Fan, Hua-Shi Guan, Chang-Yun Wang

**Affiliations:** 1 Key Laboratory of Marine Drugs, The Ministry of Education of China, School of Medicine and Pharmacy, Ocean University of China, Qingdao 266003, China; xinlanting1993@163.com (L.-T.X.); liu_qd@yahoo.com (L.L.); shaochanglun@163.com (C.-L.S.); rileiyu2010@hotmail.com (R.-L.Y.); Chenfangling0410@hotmail.com (F.-L.C.); shijun_yue@163.com (S.-J.Y.); caoyuxiaowu@163.com (M.W.); 15726227761@163.com (Z.-L.G.); fanyachu@163.com (Y.-C.F.); 2 Laboratory for Marine Drugs and Bioproducts, Qingdao National Laboratory for Marine Science and Technology, Qingdao 266071, China

**Keywords:** virtual screening, molecular docking, Topo I inhibitor, low toxic, natural product

## Abstract

Currently, DNA topoisomerase I (Topo I) inhibitors constitute a family of antitumor agents with demonstrated clinical effects on human malignancies. However, the clinical uses of these agents have been greatly limited due to their severe toxic effects. Therefore, it is urgent to find and develop novel low toxic Topo I inhibitors. In recent years, during our ongoing research on natural antitumor products, a collection of low cytotoxic or non-cytotoxic compounds with various structures were identified from marine invertebrates, plants, and their symbiotic microorganisms. In the present study, new Topo I inhibitors were discovered from low cytotoxic and non-cytotoxic natural products by virtual screening with docking simulations in combination with bioassay test. In total, eight potent Topo I inhibitors were found from 138 low cytotoxic or non-cytotoxic compounds from coral-derived fungi and plants. All of these Topo I inhibitors demonstrated activities against Topo I-mediated relaxation of supercoiled DNA at the concentrations of 5–100 µM. Notably, the flavonoids showed higher Topo I inhibitory activities than other compounds. These newly discovered Topo I inhibitors exhibited structurally diverse and could be considered as a good starting point for the development of new antitumor lead compounds.

## 1. Introduction

DNA topoisomerase I (Topo I) is a crucial enzyme that works to relax supercoiled DNA during replication, transcription, and mitosis [1,2]. In a number of human solid tumors, the intracellular level of Topo I is higher than that in normal tissues, signifying that controlling the Topo I level is essential in treating cancers [3]. Topo I inhibitors exert their antitumor activities by stabilizing the cleavable Topo I–DNA ternary complex, blocking rejoining of the DNA breaks, and inhibiting enzyme binding to DNA [4,5]. Therefore, Topo I has been considered as a promising target for the development of novel cancer chemotherapeutics [6,7,8]. Based on the mechanisms of interference with Topo I activity, these Topo I inhibitors can be grouped in two categories: Topo I poisons and Topo I catalytic inhibitors [9].

**Figure 1:**
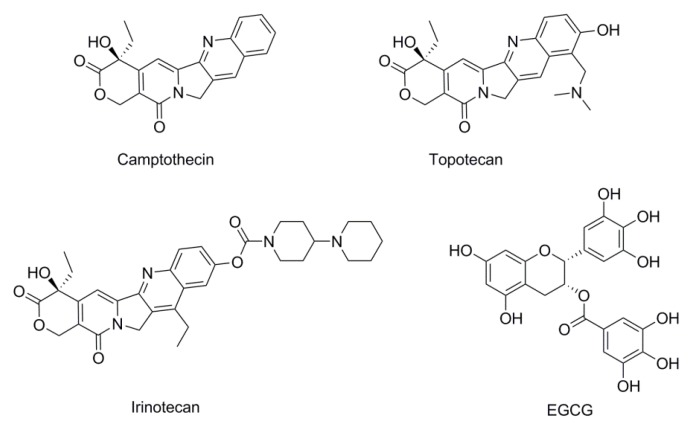
Chemical structures of representative DNA topoisomerase I (Topo I) inhibitors.

To date, a large number of Topo-directed agents (e.g., camptothecin (CPT), topotecan, and irinotecan—Figure 1) are known which are currently in clinical use [10,11]. However, their utilities are limited due to the fact that they induce severe toxic side effects such as myelosuppression, nausea, hair loss, congestive heart failure, and in some cases, increase the risk of secondary malignancies [12,13]. Recently, epigallocatechin-3-gallate (EGCG)—a major polyphenolic constituent in green tea—has received much attention as a potential cancer chemopreventive agent with Topo I inhibitory activity (Figure 1) [14,15,16]. At physiologically attainable concentrations, EGCG exerts growth inhibitory effects on several human tumor cell lines, without affecting normal cell lines, resulting in a dose-dependent inhibition of cell growth [17]. Notably, EGCG possessed low cytotoxicity with much higher half maximal inhibitory concentration (IC_50_) to human tumor cell lines than the traditional Topo-directed agents [16]. Therefore, low cytotoxic compounds may have the potential with Topo I inhibitory activity and provide the possibility for searching for novel, nontoxic Topo I inhibitors.

So far, the discovery of novel Topo I inhibitors has been facilitated by the improvement of a variety of biochemical and cellular assays, as well as molecular docking based on X-ray crystal structures [18,19,20]. Molecular docking is an application to predict how a protein interacts with small molecules. Based on the docking simulations, virtual screening has become a powerful tool for the discovery of Topo I inhibitors.

In our previous studies, hundreds of antitumor natural products have been isolated from marine invertebrates, plants, and their symbiotic microorganisms [21,22,23,24]. During the course of discovering antitumor compounds, a collection of natural products with low cytotoxic or non-cytotoxic activity were also identified. In the present study, from these low cytotoxic and non-cytotoxic natural products, Topo I inhibitors were discovered based on virtual screening with docking simulations in combination with bioassay test. By this approach, eight potent Topo I inhibitors with low cytotoxic or non-cytotoxic activity were found from the natural products isolated from coral-derived fungi and plants.

## 2. Results and Discussion

In our previous studies, hundreds secondary metabolites were isolated from marine invertebrates, plants, and their symbiotic microorganisms. Among them, there are a number of compounds exhibiting low cytotoxicity or non-cytotoxicity. In this study, 138 compounds (Table S1) from coral-derived fungi and plants with low cytotoxic and non-cytotoxic activity were selected for the screening of Topo I inhibitors by virtual screening combined with bioassay test.

### 2.1. Virtual Screening

**Figure 2:**
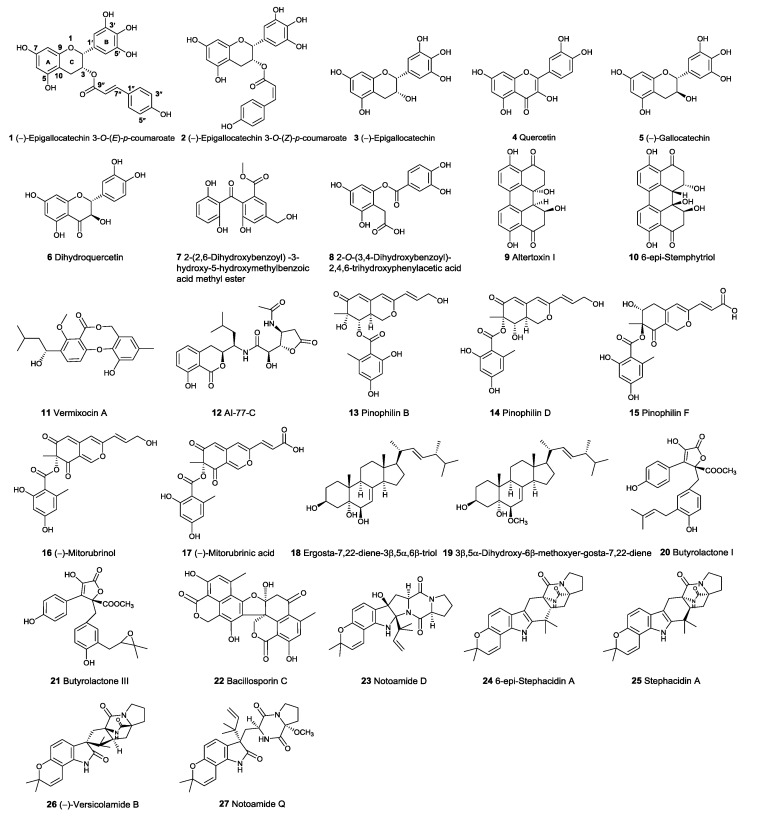
Structures of the potential active compounds from virtual screening.

To determine whether the low toxic compounds have potential as Topo I inhibitors, a total of 138 selected compounds were docked into the central catalytic domain of the Topo I–DNA complex (PDB ID: 1K4T) by using molecular operating environment (MOE) program. The docking score at −9.0 kcal/mol was used as a cutoff value for the selection of initial compounds. Thus, the 61 top-ranked complexes were first selected. Then, the selected molecules were further screened based on the following criteria: (1) Complementarity exists between the ligand and the active site of Topo I; (2) Reasonable chemical structures and conformations are in the active site of Topo I. Some unusually highly scored molecules, such as those containing a long aliphatic moiety with many rotatable bonds, were excluded for further evaluation; (3) There is a formation of hydrogen bonds between the ligand and the important residues of Topo I, such as Arg364, Asp533, and Asn722 [25]; (4) The binding mode of the compounds can be reproduced by the LeDock program (cutoff value at −5.0 kcal/mol). As a result, only 27 compounds met the above criteria (Figure 2).

### 2.2. DNA Topo I Inhibitory Activity Assay

**Figure 3:**
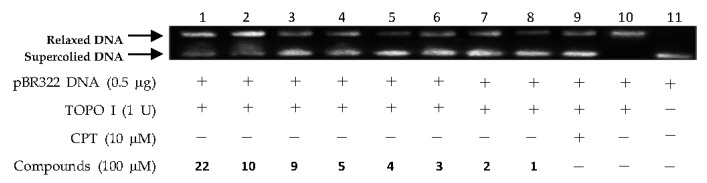
DNA Topo I inhibitory activities of (−)-epigallocatechin 3-*O*-(*E*)-*p*-coumaroate (**1**), (x)-epigallocatechin 3-*O*-(*Z*)-*p*-coumaroate (**2**), (−)-epigallocatechin (**3**), quercetin (**4**), (−)-gallocatechin (**5**), altertoxin I (**9**), 6-epi-stemphytriol (**10**), and bacillosporin C (**22**) at 100 µM. Lanes 1–8: DNA + Topo I + tested compounds; lane 9: DNA + Topo I + CPT; lane 10: DNA + Topo I; lane 11: DNA.

**Figure 4:**
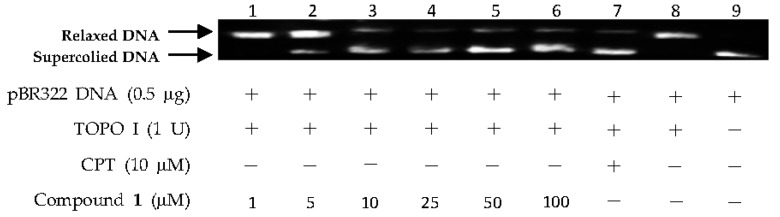
DNA Topo I inhibitory activity of (−)-epigallocatechin 3-*O*-(*E*)-*p*-coumaroate (**1**) at various concentrations (1, 5, 10, 25, 50, and 100 µM). Lanes 1–6: DNA + Topo I + compound **1** at various concentrations; lane 7: DNA + Topo I + camptothecin (CPT); lane 8: DNA + Topo I; lane 9: DNA.

**Figure 5:**
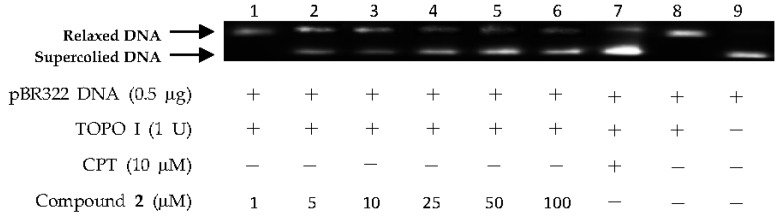
DNA Topo I inhibitory activity of (−)-epigallocatechin 3-*O*-(*Z*)-*p*-coumaroate (**2**) at various concentrations (1, 5, 10, 25, 50 and 100 µM). Lanes 1–6: DNA + Topo I + compound **2** at various concentrations; lane 7: DNA + Topo I + CPT; lane 8: DNA + Topo I; lane 9: DNA.

**Figure 6:**
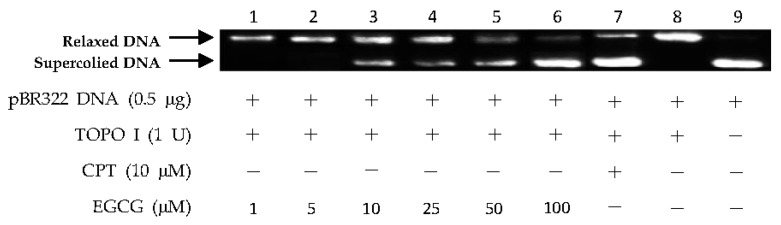
DNA Topo I inhibitory activity of epigallocatechin-3-gallate (EGCG) at various concentrations (1, 5, 10, 25, 50, and 100 µM). Lanes 1–6: DNA + Topo I + EGCG at various concentrations; lane 7: DNA + Topo I + CPT; lane 8: DNA + Topo I; lane 9: DNA.

The above virtual screening results were confirmed by Topo I inhibitory activity assay. Inhibition of the catalytic activity of Topo I has been a useful strategy for the discovery of potential antitumor agents. Topo I creates transient breaks in supercoiled DNA, resulting in DNA relaxation. The relaxed DNA can be distinguished from supercoiled DNA by gel electrophoresis analysis. In the present study, the Topo I inhibitor activities of the selected compounds were detected by monitoring the relaxation of supercoiled DNA by Topo I. Eight of the 27 compounds by virtual screening were discovered to be active against Topo I-mediated relaxation of supercoiled DNA at the concentration of 100 µM (Figure 3). The Topo I inhibition activity of eight hits were further tested at lower concentrations. Among them, four compounds—(−)-epigallocatechin 3-*O*-(*E*)-*p*-coumaroate (**1**), (−)-epigallocatechin 3-*O*-(*Z*)-*p*-coumaroate (**2**), (−)-epigallocatechin (**3**), and quercetin (**4**)—showed activity at 25 µM, and two compounds (**1** and **2**) exhibited activity at 5 µM (Figure 4 and Figure 5). It should be pointed out that compounds **1** and **2** displayed higher inhibitiory activity than EGCG (10 µM) (Figure 6).

In addition, it should be noted that flavonoids showed higher Topo I inhibitory activities than other compounds. The structure–activity relationship (SAR) analysis of these flavonoids revealed that: (1) (−)-epigallocatechin 3-*O*-(*E*)-*p*-coumaroate (**1**) and (−)-epigallocatechin 3-*O*-(*Z*)-*p*-coumaroate (**2**) with a *p*-hydroxy-cinnamic acid group at the position of C-3 could increase the inhibitory activity, while other substitution patterns might not have favorable effects on the activity; (2) the presence of double bonds between C-2 and C-3 as in quercetin (**4**) could enhance the inhibitory activity; (3) the exist of a trihydroxy moiety at the B ring might also improve the activity.

### 2.3. Binding Mode of the Representative New Topo I Inhibitors

**Figure 7:**
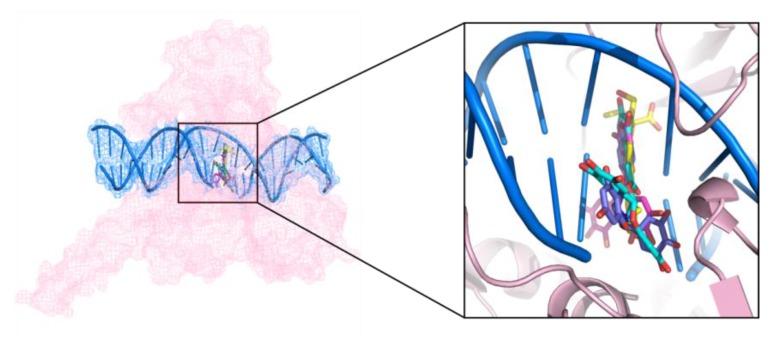
Intercalation of topotecan (yellow), EGCG (pink), (−)-epigallocatechin 3-*O*-(*E*)-*p*-coumaroate (blue), and (−)-epigallocatechin 3-*O*-(*Z*)-*p*-coumaroate (purple) in the Topo I active site.

**Figure 8:**
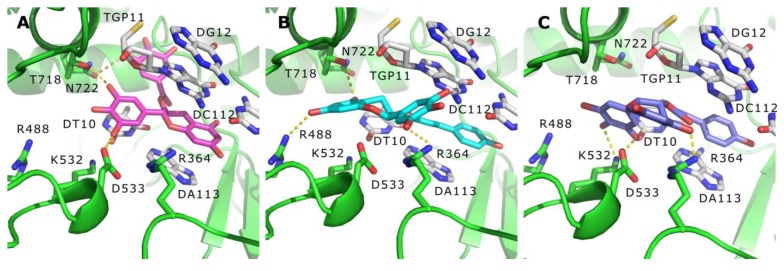
Detailed docked views of different compounds: EGCG (**A**), (−)-epigallocatechin 3-*O*-(*E*)-*p*-coumaroate (**B**), and (−)-epigallocatechin 3-*O*-(*Z*)-*p*-coumaroate (**C**).

Among the identified hits, (−)-epigallocatechin 3-*O*-(*E*)-*p*-coumaroate (**1**) and (−)-epigallocatechin 3-*O*-(*Z*)-*p*-coumaroate (**2**)—a pair of isomers—showed the most potent activities in the Topo I inhibition assay. These two compounds have structures similar to that of EGCG, and all of them belong to epigallocatechin. The bonding mode of these representative Topo I inhibitors were observed on the PyMol. In the active site cavity, the orientations of these three compounds were perpendicular to the main axis of the DNA, similar to the known Topo I inhibitors, topotecan, and paralleled to the bases (Figure 7), forming base stacking interactions with the surrounding base pairs (Figure 8). In addition, they could form hydrogen-bonding interactions with the surrounding residues. For example, at the structures of these three flavonoids, the hydroxyl oxygen atoms at the B ring could form hydrogen bonds with the residues of Arg364, Asp533, and Thr718 (Figure 8), resulting in the improvement of binding affinity.

Although the three flavonoids—(−)-epigallocatechin 3-*O*-(*E*)-*p*-coumaroate (**1**), (−)-epigallocatechin 3-*O*-(*Z*)-*p*-coumaroate (**2**), and EGCG—could bind with the Topo I active sites, EGCG shared a different conformation in the active site of Topo I (Figure 8). The benzopyrone moiety of EGCG could deeply intercalate at the DNA cleavage site and stack with the base pairs. Different from EGCG (Figure 8A), in compound **1**, the 5-hydroxyloxygen atom and 7-hydroxyloxygen atom at benzopyrone moiety could form hydrophobic interactions with the surrounding hydrophobic residues, Asn 722 and Arg 488, respectively (Figure 8B). Additionally, the hydrogen bonding interaction was also observed between the 5-hydroxyloxygen atom of compound **2** and Arg364 (Figure 8C). In summary, compounds **1**, **2**, and EGCG have three common features binding to Topo I–DNA complex: (1) a planar aromatic ring could intercalate at the DNA cleavage site; (2) base stacking interactions could form between the ligands and the base pairs; (3) at least three hydrogen bonds could be formed between the ligand and the important residues of Topo I, such as Arg364, Asp533, and Thr718.

## 3. Materials and Methods

### 3.1. General Experimental Procedures

Electrophoresis apparatus DYY-8C (Beijing Liuyi Biotechnology Co., Ltd., Beijing, China) was used for electrophoresis analysis. Gel imaging system JS-680B (Shanghai Peiqing Science and Technology Co., Ltd., Shanghai, China) was used for observation of DNA strips. Cooling and heating block CHB-100 (Hangzhou Bioer Technology Co., Ltd., Hangzhou, China) was used for the reaction of DNA and Topo I. Calf thymus Topo I and supercoiled pBR322 plasmid DNA were purchased from Takara Biotechnology Company (Dalian, China). Camptothecin (CPT, 98%) and epigallocatechin-3-gallate (EGCG, 98%) were purchased from Shanghai Aladdin Industrial Corporation (Shanghai, China) and used as positive controls. Dimethyl sulfoxide (DMSO) was purchased from Tianjin Guancheng Chemical Reagent Co., Ltd. (Tianjin, China) and used as solvent.

### 3.2. Molecular Docking

Molecular docking was used for virtual screening of the selected compounds. The MOE Dock (version 2014. 0901, Chemical Computing Group Inc., Tokyo, Japan) and the LeDock (version 1.0, http://lephar.com/) were operated to dock the compounds into the active sites of the known antitumor target protein. The molecular mechanic force field of MOE Dock used in molecular docking was set as AMBER10: EHT. Two rescoring functions, including London dG and GBVI/WSA dG, were used for pose scoring [26].

The X-ray crystallographic of Topo I–DNA in complex with topotecan (PDB ID: 1K4T) was downloaded from the Protein Data Bank (PDB, http://www.rcsb.org/) and used as a reference model for molecular docking [19]. Suitable protonation of the protein was executed at physiological pH. By using MOE software (version 2014. 0901, Chemical Computing Group Inc, Tokyo, Japan) at the AMBER10: EHT force field, water molecules were removed, hydrogen atoms were added, and energy was minimized [27].

The structures of the ligands were generated in the cdx format using the ChemBio Draw Ultra (version 14.0, PerkinElmer Inc., Fremont, CA, USA). These ligands were converted to the mol2 format and the structures were optimized by the function of minimize energy in ChemBio 3D Ultra (version 14.0, PerkinElmer Inc., Fremont, CA, USA). Further optimizations of the structures of these molecules were made by the energy minimization module in MOE software. During energy minimization by MOE software, the AMBER10 force field was used. Energy minimization was converged when the energy gradient reached 0.01 kcal/mol/Å^3^. All of the ligands used for the docking studies were assigned to suitable protonation status corresponding to physiological pH [27].

### 3.3. Preparation of the Tested Compounds

From the secondary metabolites isolated from marine invertebrates, plants, and their symbiotic microorganisms in our lab, 138 compounds (Table S1) with low cytotoxic or non-cytotoxic activity isolated from coral-derived fungi and plants were selected for virtual screening and DNA Topo I inhibition assay. For the bioassay, the tested compounds were firstly dissolved in DMSO, and then diluted with DMSO to obtain a serial solution with the concentrations of 100, 50, 25, 10, 5, and 1 µM. EGCG was also dissolved with the concentrations of 100, 50, 25, 10, 5, and 1 µM. The positive control CPT was prepared at the concentration of 10 µM.

### 3.4. DNA Topoisomerase I Inhibitory Activity Assay

The Topo I inhibitory activity was measured by assessing the relaxation of supercoiled pBR322 plasmid DNA. The reaction mixture (20 µL each), containing 35 mM Tris-HCI (pH 8.0), 72 mM KCI, 5 mM MgCl_2_, 5 mM dithiothreitol (DTT), 5 mM spermidine, 0.01% bovine serum albumin (BSA), 0.5 µg pBR322 plasmid DNA, 1.0 U calf thymus DNA Topo I, and 0.2 µL various concentrations of tested compounds, were incubated at 37 °C for 30 min. The reactions were terminated by adding dye solution containing 1% SDS, 0.02% bromophenol blue, and 50% glycerol. The mixtures were applied to 1% agarose gel and subjected to electrophoresis for 1 h in Tris-borate-EDTA buffer (0.089 mM). The gel was stained with Gelred and visualized under UV illumination and then photographed with a Gel imaging system.

## 4. Conclusions

In the present study, Topo I inhibitors were discovered from natural products with low cytotoxic and non-cytotoxic activity by virtual screening with docking simulations in combination with bioassay test. The 27 compounds with potential Topo I inhibition activity were screened from 138 marine and plant-derived natural products by means of virtual screening. On the basis of virtual screening, eight active compounds were discovered through the verification approach by bioassay. All of these Topo I inhibitors were found to be active against Topo I-mediated relaxation of supercoiled DNA at the concentrations of 5–100 µM. Notably, the flavonoids showed higher Topo I inhibitory activities than other compounds. The above results suggested that the low cytotoxic or non-cytotoxic compounds might possess Topo I inhibitory activities, and have value to be further studied for the rational drug design of antitumor agents.

## Supplementary Materials

The following are available online at http://www.mdpi.com/1660-3397/15/7/217/s1. Table S1: 138 compounds derived from coral-derived fungi and plants; Table S2: The binding energy of 138 compounds bound with the crystal structure of the ternary complex of topotecan-DNA-Topo I (PDB ID: 1K4T); Figure S1: DNA Topo I inhibitory activities of (−)-epigallocatechin 3-*O*-(*E*)-*p*-coumaroate (**1**), (−)-epigallocatechin 3-*O*-(*Z*)-*p*-coumaroate (**2**), (−)-epigallocatechin (**3**), quercetin (**4**) and altertoxin I (**9**) at 50 µM; Figure S2: DNA Topo I inhibitory activities of (−)-epigallocatechin 3-*O*-(*E*)-*p*-coumaroate (**1**), (−)-epigallocatechin 3-*O*-(*Z*)-*p*-coumaroate (**2**), (−)-epigallocatechin (**3**), quercetin (**4**) and altertoxin I (**9**) at 25 µM; Figure S3: DNA Topo I inhibitory activities of (−)-epigallocatechin 3-*O*-(*E*)-*p*-coumaroate (**1**) and (−)-epigallocatechin 3-*O*-(*Z*)-*p*-coumaroate (**2**) at 10, 5 and 1 µM; Figure S4: DNA Topo I inhibitory activities of (−)-epigallocatechin (**3**) and quercetin (**4**) at 10, 5 and 1 µM.
