# Supplementary material for: Discovery of DNA Topoisomerase I Inhibitors with Low-Cytotoxicity Based on Virtual Screening from Natural Products
Source: Mar Drugs. 2017 Jul 9;15(7):217. doi: 10.3390/md15070217 (PMC5532659; doi:10.3390/md15070217)
Supplement: Supplementary file 1 [file marinedrugs-15-00217-s001.pdf]

Supplementary Materials

Discovery of DNA topoisomerase I inhibitors with low-cytotoxicity based on virtual screening from natural products

Lan-Ting Xin[1,2 †], Lu Liu[1,2 †], Chang-Lun Shao[1,2], Ri-Lei Yu[1,2], Fang-Ling Chen[1,2], Shi-Jun Yue[1,2], Mei Wang[1,2], Zhong-Long Guo[1,2], Ya-Chu Fan[1,2], Hua-Shi Guan[1,2 *], Chang-Yun Wang[1,2 *]

[1] Key Laboratory of Marine Drugs, The Ministry of Education of China, School of Medicine and Pharmacy, Ocean University of China, Qingdao 266003, P. R. China; xinlanting1993@163.com (L.-T. X.); liu_qd@yahoo.com (L. L); shaochanglun@163.com (C.-L. S.); rileiyu2010@hotmail.com (R.-L. Y.); Chenfangling0410@hotmail.com (F.-L. C.); shijun_yue@163.com (S.-J. Y.); caoyuxiaowu@163.com (M. W.); 15726227761@163.com (Z.-L. G.); fanyachu@163.com (Y.-C. F.)

[2] Laboratory for Marine Drugs and Bioproducts, Qingdao National Laboratory for Marine Science and Technology, Qingdao 266071, P. R. China

* Correspondence: changyun@ouc.edu.cn (C.-Y. W.); hsguan@ouc.edu.cn (H.-S. G.)
Tel.: +86 532 82031536 (C.-Y. W.); +86 532 82031667 (H.-S. G.)

† These authors contributed equally to this work.

List of Supplementary Materials

Table S1 138 compounds derived from coral-derived fungi and plants.

Table S2 The binding energy of 138 compounds bound with the crystal structure of the ternary complex of topotecan-DNA-Topo I.

Figure S1 DNA Topo I inhibitory activities of (−)-epigallocatechin 3-*O*-(*E*)-*p*-coumaroate (**1**), (−)-epigallocatechin 3-*O*-(*Z*)-*p*-coumaroate (**2**), (−)-epigallocatechin (**3**), quercetin (**4**) and altertoxin I (**9**) at 50 μM.

Figure S2 DNA Topo I inhibitory activities of (−)-epigallocatechin 3-*O*-(*E*)-*p*-coumaroate (**1**), (−)-epigallocatechin 3-*O*-(*Z*)-*p*-coumaroate (**2**), (−)-epigallocatechin (**3**), quercetin (**4**) and altertoxin I (**9**) at 25 μM.

Figure S3 DNA Topo I inhibitory activities of (−)-epigallocatechin 3-*O*-(*E*)-*p*-coumaroate (**1**) and (−)-epigallocatechin 3-*O*-(*Z*)-*p*-coumaroate (**2**) at 1, 5, and 10 μM.

Figure S4 DNA Topo I inhibitory activities of (−)-epigallocatechin (**3**) and quercetin (**4**) at 1, 5, and 10 μM.

Table S1 138 compounds derived from coral-derived fungi and plants.

Compounds	Sources	Structures	Low/non-cytotoxicity[a]
S-(−)-5,6,8-Trihydroxy-4-(1′-hydroxyethyl)isocoumarin	Gorgonian-derivd fungi *Penicillium purpurogenum*	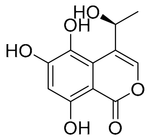	Ref. [1]: HL-60: no cytotoxicity; K562: no cytotoxicity; A549: no cytotoxicity.
Sescandelin B	Gorgonian-derivd fungi *Penicillium purpurogenum*	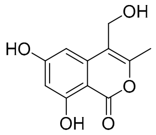	Ref. [2]: CF-1: weak cytotoxicity at 2 μg/ml.
Penicimarin E	Gorgonian-derivd fungi *Penicillium purpurogenum*	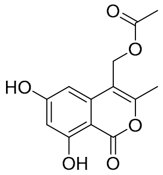	Ref. [1]: HL-60: no cytotoxicity; K562: no cytotoxicity; A549: no cytotoxicity.
8-Hydroxy-6-methoxy-3-methylisocoumarin	Gorgonian-derivd fungi *Penicillium purpurogenum*	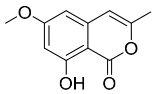	Ref. [3]: HCT-8: $IC_{50} > 25$ μg/ml; MDA-MB-435: $IC_{50} > 25$ μg/ml; SF295: $IC_{50} > 25$ μg/ml; HL-60: $IC_{50} > 25$ μg/ml.
Penicimarin B	Gorgonian-derivd fungi *Penicillium purpurogenum*	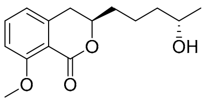	Ref. [1]: HL-60: no cytotoxicity; K562: no cytotoxicity; A549: no cytotoxicity.
N-(4-Hydroxy-2-methoxyphenyl)acetamide	Gorgonian-derivd fungi *Penicillium purpurogenum*	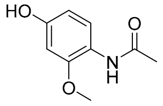	Our test: Hela: no cytotoxicity; RD: no cytotoxicity; Hep-2: no cytotoxicity; A549: no cytotoxicity.

4-Acetoxy-2-methoxyacetanilide	Gorgonian-derivd fungi<br>*Penicillium purpurogenum*	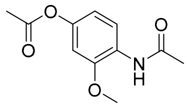	Our test:<br>Hela: no cytotoxicity;<br>RD: no cytotoxicity;<br>Hep-2: no cytotoxicity;<br>A549: no cytotoxicity.
S-(−)-2-(2-Hydroxypropanamido)benzamide	Gorgonian-derivd fungi<br>*Penicillium purpurogenum*	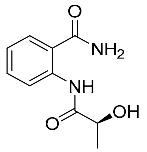	Our test:<br>Hela: no cytotoxicity;<br>RD: no cytotoxicity;<br>Hep-2: no cytotoxicity;<br>A549: no cytotoxicity.
Nafuredin	Gorgonian-derivd fungi<br>*Penicillium purpurogenum*	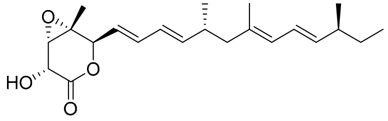	Ref. [4]:<br>L5178Y: no cytotoxicity.
2-Methoxybenzene-1,4-diol	Gorgonian-derivd fungi<br>*Penicillium purpurogenum*	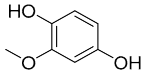	Our test:<br>Hela: $IC_{50} > 10$ µM;<br>RD: $IC_{50} > 10$ µM ;<br>Hep-2: $IC_{50} > 10$ µM ;<br>A549: $IC_{50} > 10$ µM.
3,4-Dimethoxyphenol	Gorgonian-derivd fungi<br>*Penicillium purpurogenum*	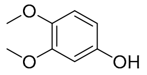	Our test:<br>Hela: no cytotoxicity;<br>RD: no cytotoxicity;<br>Hep-2: no cytotoxicity;<br>A549: no cytotoxicity.
Phenyl-acetic acid methyl ester	Gorgonian-derivd fungi<br>*Penicillium purpurogenum*	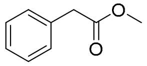	Our test:<br>Hela: no cytotoxicity;<br>RD: no cytotoxicity;<br>Hep-2: no cytotoxicity;<br>A549: no cytotoxicity.
Metyl *p*-hydroxy phenyl acetate	Gorgonian-derivd fungi<br>*Penicillium purpurogenum*	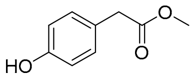	Our test:<br>Hela: no cytotoxicity;<br>RD: no cytotoxicity;<br>Hep-2: no cytotoxicity;<br>A549: no cytotoxicity.
3-Methoxy phenol	Gorgonian-derivd fungi<br>*Penicillium purpurogenum*	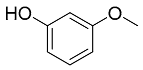	Our test:<br>Hela: no cytotoxicity;<br>RD: no cytotoxicity;<br>Hep-2: no cytotoxicity;

			A549: no cytotoxicity.
Penicillixanthone A	Gorgonian-derived fungi *Talaromyces stipitatus*	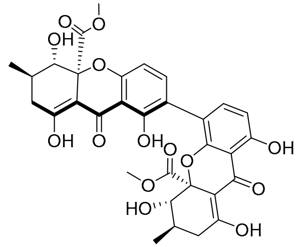	Our test:<br>K562: IC_{50} > 10 µM;<br>Vero: IC_{50} > 10 µM.
Versixanthone F	Gorgonian-derived fungi *Talaromyces stipitatus*	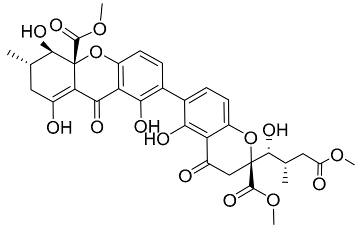	Ref. [5]:<br>A549: IC_{50} = 10.6 µM;<br>HO9810: IC_{50} = 20.8 µM.
Bacillosporin C	Gorgonian-derivd fungi *Talaromyces stipitatus*	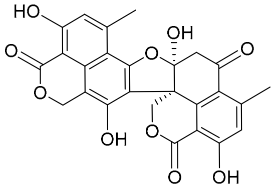	Our test:<br>HepG2: IC_{50} > 10 µM;<br>HCT-116: IC_{50} > 10 µM;<br>HL-60: IC_{50} > 10 µM;<br>A549: IC_{50} > 10 µM.
Vermixocin A	Gorgonian-derived fungi *Talaromyces stipitatus*	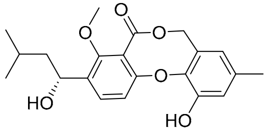	Ref. [6]:<br>P388: no cytotoxicity at 25 µg/ml.
ω-Hydroxyemodin	Gorgonian-derived fungi *Talaromyces stipitatus*	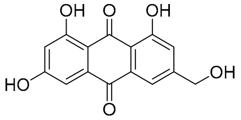	Ref. [7]:<br>MCF-7: IC_{50} = 22 µg/ml;<br>NCI-H187: IC_{50} = 39 µg/ml;<br>Vero: IC_{50} > 50 µg/ml.

2-(2,6-Dihydroxybenzoyl)-3-hydroxy-5-hydroxymethylbenzoic acid methyl ester	Gorgonian-derived fungi<br>*Talaromyces stipitatus*	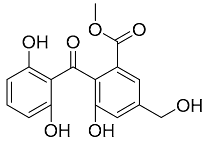	Our test:<br>K562: no cytotoxicity;<br>Hela: IC_{50} > 10 μM;<br>HCT-116: IC_{50} > 10 μM;<br>A549: IC_{50} > 10 μM.
3-Hydroxymethyl-6,8-dihydroxyisocoumarin	Gorgonian-derived fungi<br>*Talaromyces stipitatus*	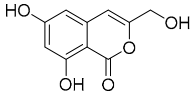	Our test:<br>Hela: IC_{50} > 10 μM;<br>Hep-2: IC_{50} > 10 μM;<br>A549: IC_{50} > 10 μM;<br>RD: IC_{50} > 10 μM.
Di-(2-ethyl)-hexylphthalate	Gorgonian-derived fungi<br>*Talaromyces stipitatus*	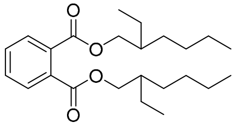	Ref. [8]:<br>NCI-H460: no cytotoxicity;<br>MCF-7: no cytotoxicity;<br>HepG2: no cytotoxicity;<br>SF268: no cytotoxicity.
Cytosporin L	Gorgonian-derived fungi<br>*Eutypella* sp.	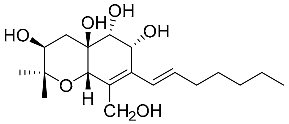	Our test:<br>K562: IC_{50} > 10 μM;<br>MCF-7: IC_{50} > 10 μM;<br>A549: IC_{50} > 10 μM;<br>Vero: IC_{50} > 10 μM.
Cytosporin E	Gorgonian-derived fungi<br>*Eutypella* sp.	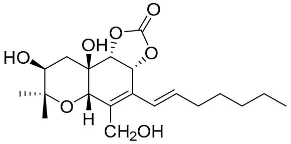	Our test:<br>K562: IC_{50} > 10 μM;<br>MCF-7: IC_{50} > 10 μM;<br>A549: IC_{50} > 10 μM;<br>Vero: IC_{50} > 10 μM.
Cytosporin D	Gorgonian-derived fungi<br>*Eutypella* sp.	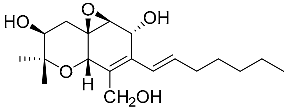	Our test:<br>K562: IC_{50} > 10 μM;<br>MCF-7: IC_{50} > 10 μM;<br>A549: IC_{50} > 10 μM;<br>Vero: IC_{50} > 10 μM.
N-Acetyltryptamine	Gorgonian-derived fungi<br>*Eutypella* sp.	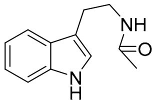	Our test:<br>K562: IC_{50} > 10 μM;<br>MCF-7: IC_{50} > 10 μM;<br>A549: IC_{50} > 10 μM;

			Vero: IC_{50} > 10 μM.
(11*R*)-2,11,12-Trihydroxy-β-selinene	Gorgonian-derived fungi *Eutypella* sp.	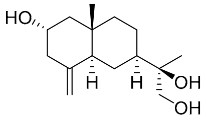	Our test:<br>K562: IC_{50} > 10 μM;<br>MCF-7: IC_{50} > 10 μM;<br>A549: IC_{50} > 10 μM;<br>Vero: IC_{50} > 10 μM.
(11*S*)-2,11,13-Trihydroxy-β-selinene	Gorgonian-derived fungi *Eutypella* sp.	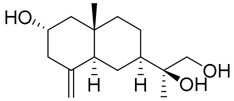	Our test:<br>K562: IC_{50} > 10 μM;<br>MCF-7: IC_{50} > 10 μM;<br>A549: IC_{50} > 10 μM;<br>Vero: IC_{50} > 10 μM.
Butyrolactone I	Gorgonian-derived fungi *Eutypella* sp.	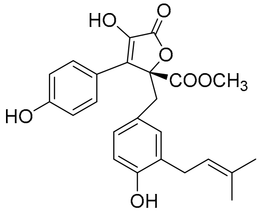	Ref. [9]:<br>MDA-MB-231: IC_{50} = 34.4 μM;<br>MCF-7: IC_{50} = 17.4 μM.
Butyrolactone II	Gorgonian-derived fungi *Eutypella* sp.	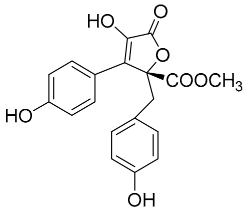	Ref. [10]:<br>HL-60: IC_{50} > 80 μM;<br>ASPC1: IC_{50} > 80 μM;<br>PC-3: IC_{50} > 80 μM;<br>HCT-116: IC_{50} > 80 μM.
Butyrolactone III	Gorgonian-derived fungi *Eutypella* sp.	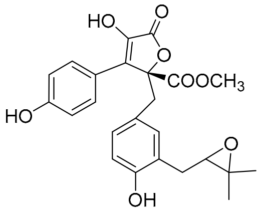	Ref. [10]:<br>HL-60: IC_{50} > 80 μM;<br>ASPC1: IC_{50} > 80 μM;<br>PC-3: IC_{50} > 80 μM;<br>HCT-116: IC_{50} > 80 μM.

Butyrolactone V	Gorgonian-derived fungi<br>*Eutypella* sp.	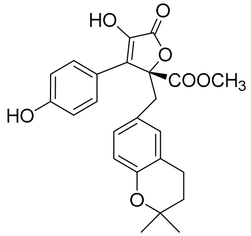	Ref. [9]:<br>MDA-MB-231: IC_{50} = 22.2 µM;<br>MCF-7: IC_{50} = 31.9 µM.
2,4-Dihydroxy-3,5,6-trimethylbenzoic acid	Gorgonian-derived fungi<br>*Eutypella* sp.	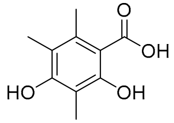	Ref. [11]:<br>HL-60: IC_{50} > 160 µM;<br>HepG2: IC_{50} > 160 µM.
Diorcinol	Gorgonian-derived fungi<br>*Eutypella* sp.	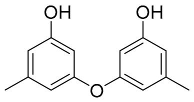	Ref. [12]:<br>SK-MEL-28: IC_{50} = 40 µM;<br>HCT-116: IC_{50} =22.8 µM.
Anodendroic acid	Gorgonian-derived fungi *Xylaria*<br>sp.	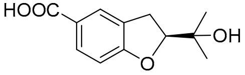	Ref [13]:<br>Hela: no cytotoxicity;<br>Hep-2: no cytotoxicity;<br>RD: no cytotoxicity;<br>A549: no cytotoxicity.
Methyl indole-3-carboxylate	Gorgonian-derived fungi *Xylaria*<br>sp.	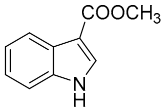	Ref [13]:<br>Hela: no cytotoxicity;<br>Hep-2: no cytotoxicity;<br>RD: no cytotoxicity;<br>A549: no cytotoxicity.
5-Formylmellein	Gorgonian-derived fungi<br>*Eutypella* sp.	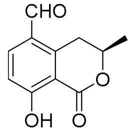	Ref [14]:<br>NUGC-3: no cytotoxicity;<br>HONE-1: no cytotoxicity.

(2*E*,4*E*,6*S*)-6-Hydroxydeca-2,4-dienoic acid	Gorgonian-derived fungi *Eutypella* sp.	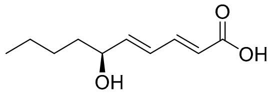	Ref [13]:<br>Hela: no cytotoxicity;<br>Hep-2: no cytotoxicity;<br>RD: no cytotoxicity;<br>A549: no cytotoxicity.
7-Chloro-5-hydroxymellein	Gorgonian-derived fungi *Xylaria* sp.	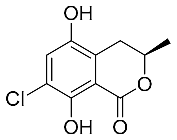	Ref [13]:<br>Hela: no cytotoxicity;<br>Hep-2: no cytotoxicity;<br>RD: no cytotoxicity;<br>A549: no cytotoxicity.
5-Carboxylmellein	Gorgonian-derived fungi *Xylaria* sp.	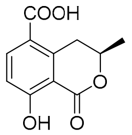	Ref [13]:<br>Hela: no cytotoxicity;<br>Hep-2: no cytotoxicity;<br>RD: no cytotoxicity;<br>A549: no cytotoxicity.
Sclerotinin A	Gorgonian-derived fungi *Eutypella* sp.	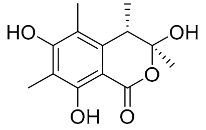	Ref [15]:<br>L5178Y: $IC_{50} > 10$ µg/ml.
Sclerotinin B	Gorgonian-derived fungi *Eutypella* sp.	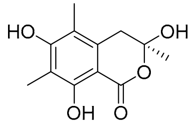	Ref [15]:<br>L5178Y: $IC_{50} > 10$ µg/ml.
Decarboxydihydrocitrinin	Gorgonian-derived fungi *Xylaria* sp.	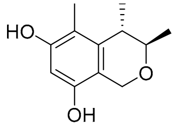	Ref [16]:<br>KB: no cytotoxicity;<br>MCF-7: no cytotoxicity;<br>Vero: no cytotoxicity.
Dihydrocitrinin	Gorgonian-derived fungi *Xylaria* sp.	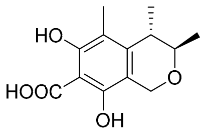	Ref [16]:<br>KB: no cytotoxicity;<br>MCF-7: no cytotoxicity;<br>Vero: no cytotoxicity.

(22*E*)-Ergosta-5,7,9(11)22-tetraen-3β-ol	Gorgonian-derived fungi *Xylaria* sp.	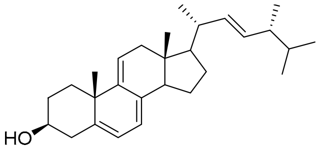	Our test:<br>Hela: no cytotoxicity;<br>Hep-2: no cytotoxicity;<br>RD: no cytotoxicity;<br>A549: no cytotoxicity.
Ergosta-5,7,22-triene-3β-ol	Gorgonian-derived fungi *Xylaria* sp.	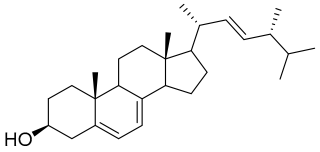	Ref [17]:<br>MCF-7: $IC_{50} > 50$ µg/ml;<br>SF-268: $IC_{50} > 50$ µg/ml;<br>NCI-H460: $IC_{50} > 50$ µg/ml.
5α,8α-Epidioxy-ergosta-6,22*E*-dien-3β-ol	Gorgonian-derived fungi *Xylaria* sp.	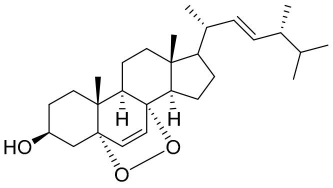	Ref [13]:<br>Hela: no cytotoxicity;<br>Hep-2: no cytotoxicity;<br>RD: no cytotoxicity;<br>A549: no cytotoxicity.
Altertoxin I	Soft coral-derived fungi *Alternaria alternata*	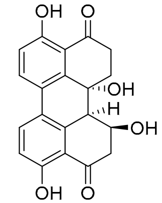	Our test:<br>HepG2: $IC_{50} > 10$ µM;<br>HCT-116: $IC_{50} > 10$ µM;<br>HL-60: $IC_{50} > 10$ µM;<br>A549: $IC_{50} > 10$ µM.
6-epi-Stemphytriol	Soft coral-derived fungi *Alternaria alternata*	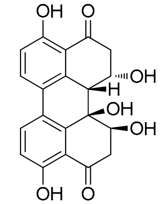	Our test:<br>Hela: $IC_{50} > 10$ µM;<br>HL-60: $IC_{50} > 10$ µM;<br>K562: $IC_{50} > 10$ µM;<br>A549: $IC_{50} > 10$ µM.

7-epi-8-Hydroxyaltertoxin I	Soft coral-derived fungi<br>*Alternaria alternata*	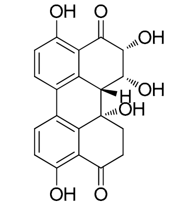	Our test:<br>Hela: $IC_{50} > 10$ µM;<br>HL-60: $IC_{50} > 10$ µM;<br>K562: $IC_{50} > 10$ µM;<br>A549: $IC_{50} > 10$ µM.
Stemphyperylenol	Soft coral-derived fungi<br>*Alternaria alternata*	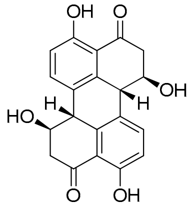	Ref [18]:<br>KB: no cytotoxicity;<br>KBv200: no cytotoxicity.
5α,8α-Epidioxy-ergosta-6,9,22*E*-trien-3β-ol	Gorgonian-derived fungi<br>*Cladosporium* sp.	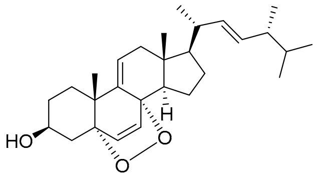	Our test:<br>Hela: no cytotoxicity;<br>RD: no cytotoxicity;<br>Hep-2: no cytotoxicity;<br>A549: no cytotoxicity.
5α,8α-Epidioxy-ergosta-6,22*E*-dien-3β-ol	Gorgonian-derived fungi<br>*Cladosporium* sp.	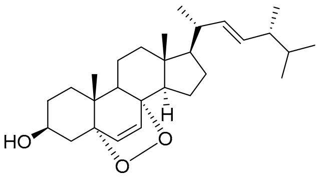	Ref [13]:<br>Hela: no cytotoxicity;<br>Hep-2: no cytotoxicity;<br>RD: no cytotoxicity;<br>A549: no cytotoxicity.
Ergosta-7,22-diene-3β,5α,6β-triol	Gorgonian-derived fungi<br>*Cladosporium* sp.	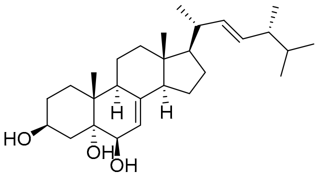	Our test:<br>Hela: $IC_{50} > 10$ µM;<br>Hep-2: $IC_{50} > 10$ µM;<br>RD: $IC_{50} > 10$ µM;<br>A549: $IC_{50} > 10$ µM;<br>Vero: $IC_{50} > 10$ µM.

3β,5α-Dihydroxy-6β-methoxyer-gosta-7,22-diene	Gorgonian-derived fungi *Cladosporium* sp.	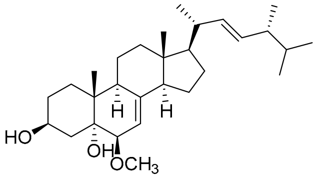	Ref [16]:<br>SK-OV-3: $IC_{50} > 30$ μM;<br>SK-MEL-2: $IC_{50} > 30$ μM;<br>XF498: $IC_{50} > 30$ μM;<br>HCT-115: $IC_{50} > 30$ μM;<br>A549: $IC_{50} > 30$ μM.
Ergosterol	Gorgonian-derived fungi *Cladosporium* sp.	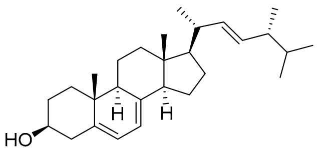	Ref [19]:<br>SK-OV-3: $IC_{50} = 12.03$ μM;<br>SK-MEL-2: $IC_{50} = 12.36$ μM;<br>XF498: $IC_{50} = 18.90$ μM;<br>HCT-115: $IC_{50} = 16.04$ μM;<br>A549: $IC_{50} = 23.11$ μM.
β-Sitosterol glucoside	Gorgonian-derived fungi *Cladosporium* sp.	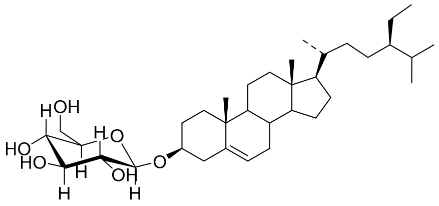	Ref [20]:<br>HCT-116: $IC_{50} = 8.52$ μg/ml.
AI-77-C	Gorgonian-derived fungi *Cladosporium* sp.	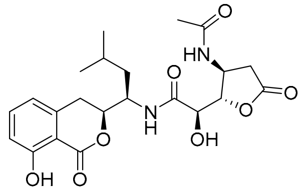	Our test:<br>Hela: $IC_{50} > 10$ μM;<br>Hep-2: $IC_{50} > 10$ μM;<br>RD: $IC_{50} > 10$ μM;<br>A549: $IC_{50} > 10$ μM.
9-Octadecenoic acid-2′,3′-dihydroxy propyl ester	Gorgonian-derived fungi *Cladosporium* sp.	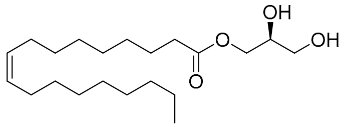	Our test:<br>Hela: no cytotoxicity;<br>RD: no cytotoxicity;<br>Hep-2: no cytotoxicity;<br>A549: no cytotoxicity.

3,4-Dihydroxybenzoic acid	Gorgonian-derived fungi<br>*Cladosporium* sp.	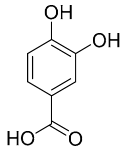	Ref [21]:<br>HL-60: IC_{50} = 64.6 μM;<br>Hel-299: IC_{50} > 100 μM.
2,3-Dihydroxybenzoic acid	Gorgonian-derived fungi<br>*Cladosporium* sp.	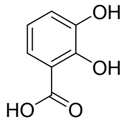	Our test:<br>Hela: no cytotoxicity;<br>HL-60: no cytotoxicity;
Indole-3-carboxylic acid	Gorgonian-derived fungi<br>*Cladosporium* sp.	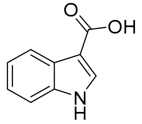	Ref [22]:<br>MCF-7: IC_{50} = 12.9 μm/ml.
6-epi-Stephacidin A	Gorgonian-derived fungi<br>*Aspergillus versicolor*	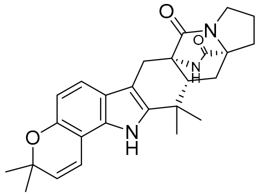	Ref [23]:<br>HL-60: no cytotoxicity;<br>A549: no cytotoxicity.
Stephacidin A	Gorgonian-derived fungi<br>*Aspergillus versicolor*	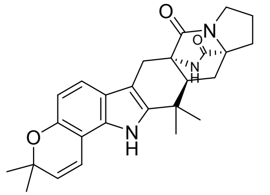	Ref [24]:<br>Hela: no cytotoxicity.
Notoamide B	Gorgonian-derived fungi<br>*Aspergillus versicolor*	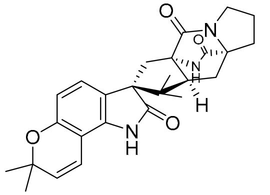	Ref [25]:<br>Hela: IC_{50} > 22 μg/ml;<br>L1210: IC_{50} >22 μg/ml.

(−)-Versicolamide B	Gorgonian-derived fungi<br>*Aspergillus versicolor*	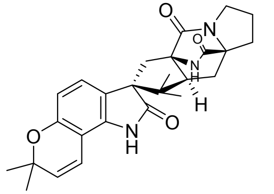	Ref [23]:<br>HL-60: no cytotoxicity;<br>A549: no cytotoxicity.
Notoamide D	Gorgonian-derived fungi<br>*Aspergillus versicolor*	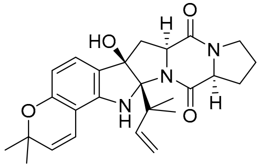	Ref [25]:<br>Hela: $IC_{50} > 100$ µg/ml;<br>L1210: $IC_{50} > 100$ µg/ml.
Notoamide Q	Gorgonian-derived fungi<br>*Aspergillus versicolor*	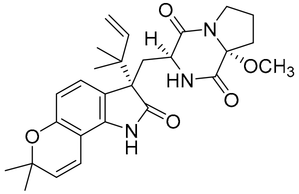	Ref [26]:<br>K562: $IC_{50} > 100$ µM;<br>ASPC: $IC_{50} > 100$ µM;<br>H460: $IC_{50} > 100$ µM;<br>A549: $IC_{50} > 100$ µM.
Kipukasin E	Gorgonian-derived fungi<br>*Aspergillus versicolor*	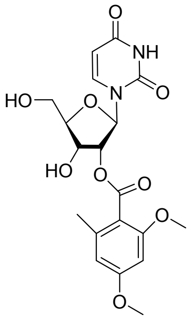	Our test:<br>K562: $IC_{50} > 10$ µM;<br>Hela: $IC_{50} > 10$ µM;<br>HCT-116: $IC_{50} > 10$ µM;<br>A549: $IC_{50} > 10$ µM.

Kipukasin D	Gorgonian-derived fungi *Aspergillus versicolor*	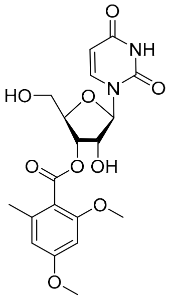	Our test: K562: $IC_{50} > 10$ μM; Hela: $IC_{50} > 100$ μM; HCT-116: $IC_{50} > 100$ μM; A549: $IC_{50} > 100$ μM.
Cordyol C	Gorgonian-derived fungi *Aspergillus versicolor*	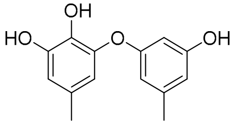	Ref [27]: K562: $IC_{50} > 10$ μM; HL-60: $IC_{50} > 10$ μM.
Diorcinol F	Gorgonian-derived fungi *Aspergillus versicolor*	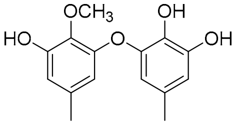	Ref [28]: PC3: no cytotoxicity; A2780: no cytotoxicity; MDA-MB-231: no cytotoxicity; A549: no cytotoxicity.
Violaceol II	Gorgonian-derived fungi *Aspergillus versicolor*	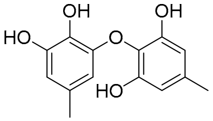	Ref [29]: KB: $IC_{50} > 10$ μm/ml; BC: $IC_{50} = 5.50$ μm/ml; NCI-H187: $IC_{50} = 3.70$ μm/ml.
Sterigmatocystin	Gorgonian-derived fungi *Aspergillus versicolor*	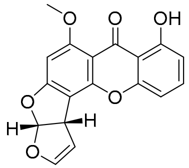	Ref [30]: L-929: $IC_{50} = 163.3$ μM; Neuro-2a: $IC_{50} = 40.1$ μM; HepG2: $IC_{50} = 286.1$ μM.

Methyl 6-acetyl-4-methoxy-5,7,8-trihydroxynaphthalene-2-carboxylate	Gorgonian-derived fungi *Aspergillus micronesiensis*	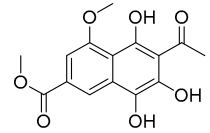	Ref [31]: K562: no cytotoxicity; Hun-7: no cytotoxicity; A549: no cytotoxicity.
3,5-Dichloroasterric acid	Gorgonian-derived fungi *Aspergillus micronesiensis*	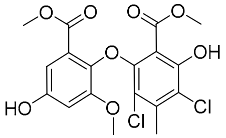	Our test: Hela: IC_{50} > 10 µM; A549: IC_{50} > 10 µM.
Penicillither	Gorgonian-derived fungi *Aspergillus micronesiensis*	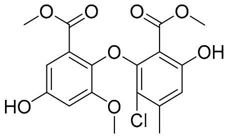	Ref [31]: KB: no cytotoxicity; MCF-7: no cytotoxicity; Vero: no cytotoxicity.
Dihydrogeodin	Gorgonian-derived fungi *Aspergillus micronesiensis*	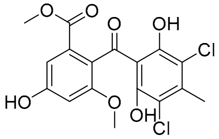	Ref [32]: KB: no cytotoxicity; MCF-7: no cytotoxicity; Vero: no cytotoxicity.
2-(3-Chloro-4-methyl-γ-resorcyloyl)-5-hydroxy-*m*-anisic acid methyl ester	Gorgonian-derived fungi *Aspergillus micronesiensis*	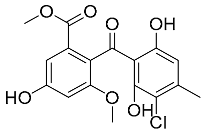	Ref [32]: KB: no cytotoxicity; MCF-7: no cytotoxicity; Vero: no cytotoxicity.
Cytochalasin Z16	Gorgonian-derived fungi *Aspergillus micronesiensis*	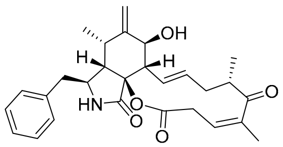	Ref [33]: HL-60: no cytotoxicity; BEL-7402: no cytotoxicity; P388: no cytotoxicity.

Cytochalasin Z17	Gorgonian-derived fungi<br>*Aspergillus micronesiensis*	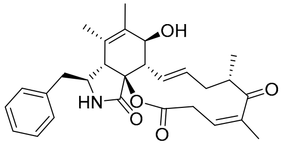	Ref [33]:<br>HL-60: no cytotoxicity;<br>BEL-7402: no cytotoxicity;<br>P388: no cytotoxicity.
9,10-Anthracenedione	Gorgonian-derived fungi<br>*Aspergillus micronesiensis*	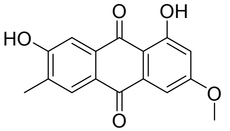	Our test:<br>L1210: $IC_{50} > 100$ μM;<br>A549: $IC_{50} > 100$ μM.
Pinophilin D	Gorgonian-derived fungi<br>*Penicillium pinophilum*	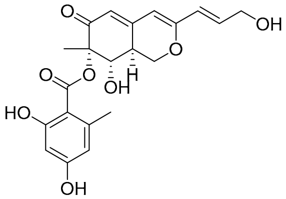	Ref [34]:<br>Hep-2: no cytotoxicity;<br>RD: no cytotoxicity;<br>Hela: no cytotoxicity.
Pinophilin E	Gorgonian-derived fungi<br>*Penicillium pinophilum*	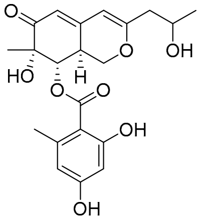	Ref [34]:<br>Hep-2: no cytotoxicity;<br>RD: no cytotoxicity;<br>Hela: no cytotoxicity.
Pinophilin F	Gorgonian-derived fungi<br>*Penicillium pinophilum*	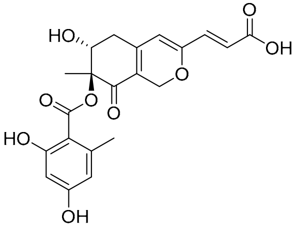	Ref [34]:<br>Hep-2: no cytotoxicity;<br>RD: no cytotoxicity;<br>Hela: no cytotoxicity.

Sch 1385568	Gorgonian-derived fungi<br>*Penicillium pinophilum*	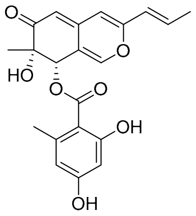	Ref [34]:<br>Hep-2: no cytotoxicity;<br>RD: no cytotoxicity;<br>Hela: no cytotoxicity.
Pinophilin B	Gorgonian-derived fungi<br>*Penicillium pinophilum*	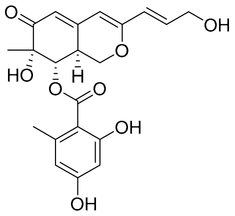	Ref [34]:<br>Hep-2: no cytotoxicity;<br>RD: no cytotoxicity;<br>Hela: no cytotoxicity.
Sch 725680	Gorgonian-derived fungi<br>*Penicillium pinophilum*	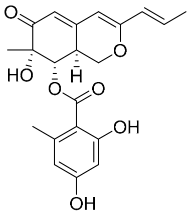	Ref [34]:<br>Hep-2: no cytotoxicity;<br>RD: no cytotoxicity;<br>Hela: no cytotoxicity.
(−)-Mitorubrin	Gorgonian-derived fungi<br>*Penicillium pinophilum*	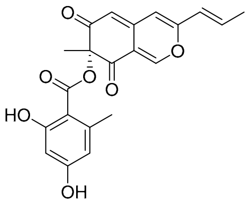	Ref [34]:<br>Hep-2: no cytotoxicity;<br>RD: no cytotoxicity;<br>Hela: no cytotoxicity.

(−)-Mitorubrinol	Gorgonian-derived fungi *Penicillium pinophilum*	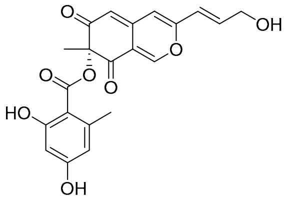	Ref [34]: Hep-2: no cytotoxicity; RD: no cytotoxicity; Hela: no cytotoxicity.
(−)-Mitorubrinic acid	Gorgonian-derived fungi *Penicillium pinophilum*	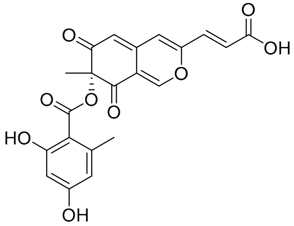	Ref [34]: Hep-2: no cytotoxicity; RD: no cytotoxicity; Hela: no cytotoxicity.
Isopenicillide	Gorgonian-derived fungi *Penicillium pinophilum*	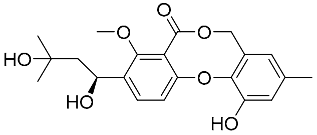	Ref [34]: Hep-2: no cytotoxicity; RD: no cytotoxicity; Hela: no cytotoxicity.
Purpactin A	Gorgonian-derived fungi *Penicillium pinophilum*	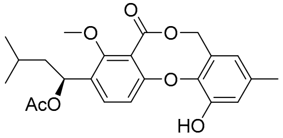	Ref [34]: Hep-2: no cytotoxicity; RD: no cytotoxicity; Hela: no cytotoxicity.
Vermistatin	Gorgonian-derived fungi *Penicillium pinophilum*	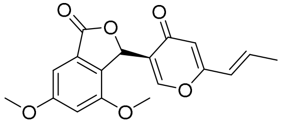	Ref [35]: KB: IC_{50} = 90.2 µm/ml; KBv200: no cytotoxicity.

Methoxyvermistatin	Gorgonian-derived fungi<br>*Penicillium pinophilum*	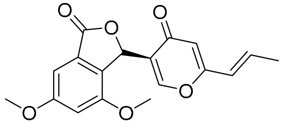	Ref [35]:<br>KB: IC_{50} = 20.0 µm/ml;<br>KBv200: IC_{50} = 15.1 µm/ml
4-Demethylvermistatin	Gorgonian-derived fungi<br>*Penicillium pinophilum*	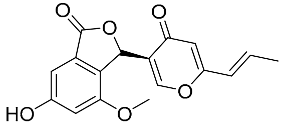	Our test:<br>Hela: no cytotoxicity;<br>Hep-2: no cytotoxicity;<br>A549: no cytotoxicity;<br>RD: no cytotoxicity.
4-Demethyl-11-methoxyvermistatin	Gorgonian-derived fungi<br>*Penicillium pinophilum*	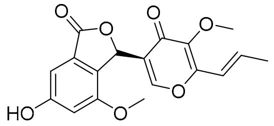	Our test:<br>Hela: no cytotoxicity;<br>Hep-2: no cytotoxicity;<br>A549: no cytotoxicity;<br>RD: no cytotoxicity.
Dihydrovermistatin	Gorgonian-derived fungi<br>*Penicillium pinophilum*	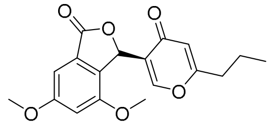	Our test:<br>Hela: no cytotoxicity;<br>Hep-2: no cytotoxicity;<br>A549: no cytotoxicity;<br>RD: no cytotoxicity.
3-*O*-Methylfunicone	Gorgonian-derived fungi<br>*Penicillium pinophilum*	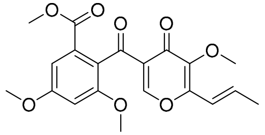	Ref [36]:<br>KB: IC_{50} = 35.3 µM.
Penifupyrone	Gorgonian-derived fungi<br>*Penicillium pinophilum*	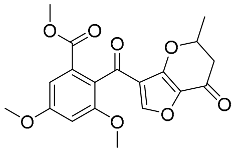	Ref [36]:<br>KB: IC_{50} = 4.7 µM.

Funicone	Gorgonian-derived fungi<br>*Penicillium pinophilum*	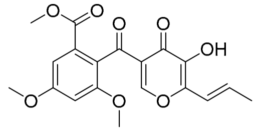	Ref [36]:<br>KB: IC_{50} = 13.2 µM.
3-*O*-Methyl-5,6-epoxyfunicone	Gorgonian-derived fungi<br>*Penicillium pinophilum*	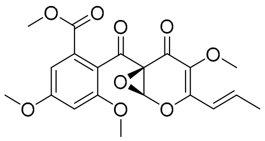	Our test:<br>Hela: no cytotoxicity;<br>Hep-2: no cytotoxicity;<br>A549: no cytotoxicity;<br>RD: no cytotoxicity.
Pestalasin A	Gorgonian-derived fungi<br>*Penicillium pinophilum*	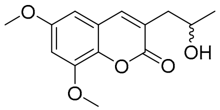	Ref [37]:<br>L5178Y: no cytotoxicity;<br>Hela: no cytotoxicity;<br>PC12: no cytotoxicity.
Cyclo(L-Leu-L-Pro)	Soft coral-derived fungi<br>*Trichoderma harzianum*	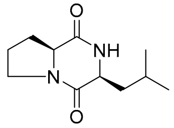	Ref [38]:<br>Hep-2: IC_{50} = 242 mg/l.
Trichoacorenol	Soft coral-derived fungi<br>*Trichoderma harzianum*	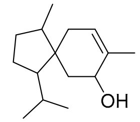	Our test:<br>MCF-7: no cytotoxicity;<br>HepG2: no cytotoxicity;<br>A549: no cytotoxicity;<br>HCT-116: no cytotoxicity.
Ascotrichic acid	Soft coral-derived fungi<br>*Trichoderma harzianum*	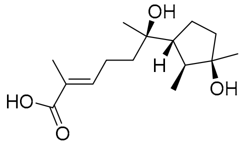	Our test:<br>MCF-7: no cytotoxicity;<br>HepG2: no cytotoxicity;<br>A549: no cytotoxicity;<br>HCT-116: no cytotoxicity.
Cyclonerotriol	Soft coral-derived fungi<br>*Trichoderma harzianum*	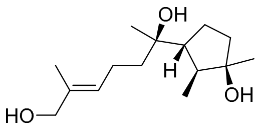	Our test:<br>MCF-7: no cytotoxicity;<br>HepG2: no cytotoxicity;<br>A549: no cytotoxicity;<br>HCT-116: no cytotoxicity.

5-Hydroxy-*N*,*N*-dimethyltryptamine	*Phyllodium pulchellum*	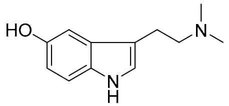	Ref [39]:<br>KB: IC_{50} > 100 mg/l;<br>HepG2: IC_{50} > 100 mg/l .
5-Hydroxy-*N*,*N*-dimethyltryptamine-oxide	*Phyllodium pulchellum*	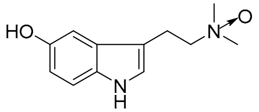	Our test:<br>Hela: IC_{50} > 10 µM;<br>Hep-2: IC_{50} > 10 µM;<br>A549: IC_{50} > 10 µM;<br>RD: IC_{50} > 10 µM.
5-Methoxy-*N*,*N*-dimethyltryptamine	*Phyllodium pulchellum*	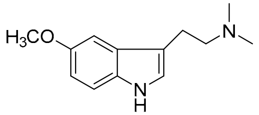	Ref [39]:<br>KB: IC_{50} > 100 mg/l;<br>HepG2: IC_{50} = 80.5 mg/l .
5-Methoxy-*N*,*N*-dimethyltryptamine-oxide	*Phyllodium pulchellum*	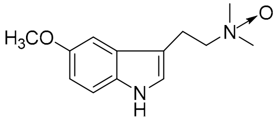	Our test:<br>Hela: IC_{50} > 10 µM;<br>Hep-2: IC_{50} > 10 µM;<br>A549: IC_{50} > 10 µM;<br>RD: IC_{50} > 10 µM.
L-Tryptophan	*Phyllodium pulchellum*	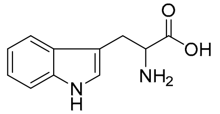	Our test:<br>Hela: IC_{50} > 10 µM;<br>Hep-2: IC_{50} > 10 µM;<br>A549: IC_{50} > 10 µM;<br>RD: IC_{50} > 10 µM.
N,*N*-Dimethyl-L-tryptophan	*Phyllodium pulchellum*	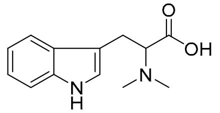	Our test:<br>Hela: IC_{50} > 10 µM;<br>Hep-2: IC_{50} > 10 µM;<br>A549: IC_{50} > 10 µM;<br>RD: IC_{50} > 10 µM.
2-(Indol-3-yl)ethyl-α-L-rhamnopyra-nosyl-(1→6)-β-D-glu copyranoside	*Phyllodium pulchellum*	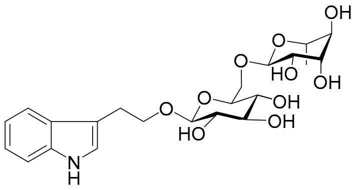	Our test:<br>Hela: IC_{50} > 10 µM;<br>Hep-2: IC_{50} > 10 µM;<br>A549: IC_{50} > 10 µM;<br>RD: IC_{50} > 10 µM.

2-(Indol-3-yl)ethyl-β-D-glucopyranoside	*Phyllodium pulchellum*	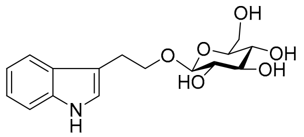	Our test:<br>Hela: $IC_{50} > 10$ μM;<br>Hep-2: $IC_{50} > 10$ μM;<br>A549: $IC_{50} > 10$ μM;<br>RD: $IC_{50} > 10$ μM.
(−)-Epigallocatechin 3-*O*-(*E*)-*p*-coumaroate	*Phyllodium pulchellum*	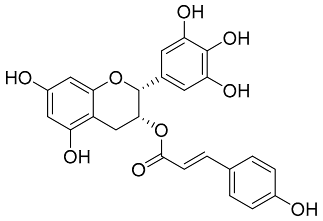	Our test:<br>Hela: $IC_{50} > 10$ μM;<br>Hep-2: $IC_{50} > 10$ μM;<br>A549: $IC_{50} > 10$ μM;<br>RD: $IC_{50} > 10$ μM.
(−)-Epigallocatechin 3-*O*-(*Z*)-*p*-coumaroate	*Phyllodium pulchellum*	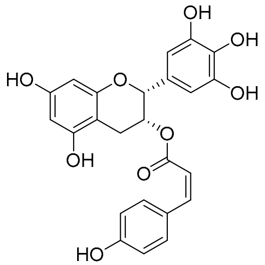	Our test:<br>Hela: $IC_{50} > 10$ μM;<br>Hep-2: $IC_{50} > 10$ μM;<br>A549: $IC_{50} > 10$ μM;<br>RD: $IC_{50} > 10$ μM.
(−)-Gallocatechin	*Phyllodium pulchellum*	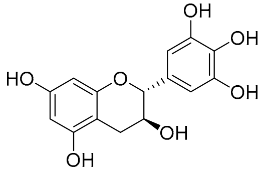	Our test:<br>Hela: $IC_{50} > 10$ μM;<br>Hep-2: $IC_{50} > 10$ μM;<br>A549: $IC_{50} > 10$ μM;<br>RD: $IC_{50} > 10$ μM.

(−)-Epigallocatechin	*Phyllodium pulchellum*	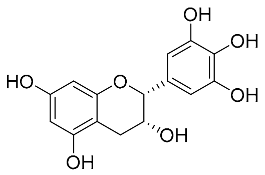	Ref [40]:<br>HSC-2: IC_{50} = 67 µM.
(+)-Catechin	*Phyllodium pulchellum*	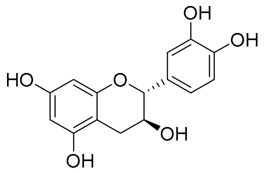	Ref [40]:<br>HSC-2: IC_{50} = 497 µM.
(−)-Epicatechin	*Phyllodium pulchellum*	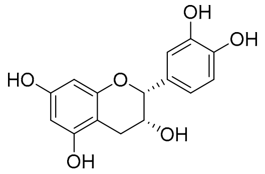	Ref [40]:<br>HSC-2: IC_{50} > 500 µM.
(+)-Dihydrokaempferol	*Phyllodium pulchellum*	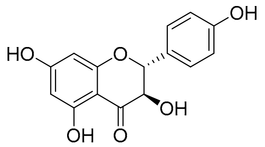	Ref [41]:<br>A-549: no cytotoxicity;<br>BEL-7402: no cytotoxicity;<br>HCT-8: no cytotoxicity;<br>A2780: no cytotoxicity;<br>BGC-823: no cytotoxicity.
Dihydroquercetin	*Phyllodium pulchellum*	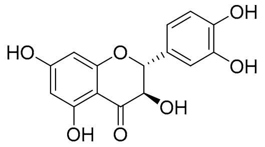	Ref [42]:<br>HCT-116: IC_{50} = 63.42 µM.

Quercetin	*Phyllodium pulchellum*	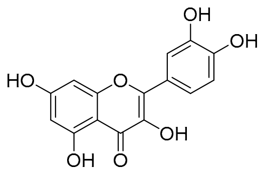	Ref [43]:<br>HT-29: IC_{50} > 30 μM;<br>Caco-2: IC_{50} > 15 μM.
Rutin	*Phyllodium pulchellum*	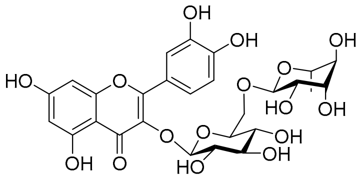	Ref [44]:<br>SW480: IC_{50} = 125 μM.
Quercetin-3-*O*-α-L-rhamnopyranosyl-(1→6)-β-D-galactopyranoside	*Phyllodium pulchellum*	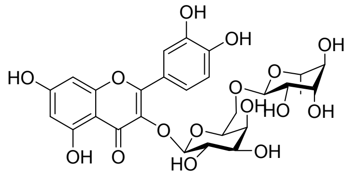	Our test:<br>Hela: IC_{50} > 10 μM;<br>Hep-2: IC_{50} > 10 μM;<br>A549: IC_{50} > 10 μM;<br>RD: IC_{50} > 10 μM.
2-*O*-(3,4-Dihydroxybenzoyl)-2,4,6-trihydroxyphenylacetic acid	*Phyllodium pulchellum*	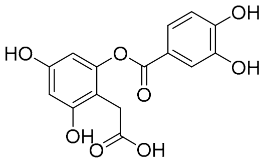	Our test:<br>Hela: IC_{50} > 100 μM;<br>Hep-2: IC_{50} > 100 μM;<br>A549: IC_{50} > 100 μM;<br>RD: IC_{50} > 100 μM.
p-Hydroxybenzoic acid	*Phyllodium pulchellum*	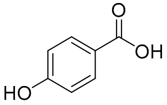	Ref [45]:<br>HepG2: no cytotoxicity;<br>Hep-3B: no cytotoxicity;<br>MCF-7: no cytotoxicity;<br>MDA-MB-231: no cytotoxicity;<br>A-549: no cytotoxicity.
Protocatechuic acid	*Phyllodium pulchellum*	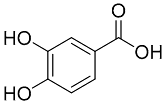	Ref [46]:<br>HL-60: IC_{50} = 4.54 mM.

Protocaechuic acid methyl ester	*Phyllodium pulchellum*	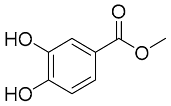	Our test:<br>Hela: IC_{50} > 10 μM;<br>Hep-2: IC_{50} > 10 μM;<br>A549: IC_{50} > 10 μM;<br>RD: IC_{50} > 10 μM.
Protocatechuic acid ethyl ester	*Phyllodium pulchellum*	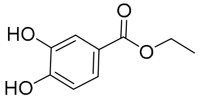	Our test:<br>Hela: IC_{50} > 10 μM;<br>Hep-2: IC_{50} > 10 μM;<br>A549: IC_{50} > 10 μM;<br>RD: IC_{50} > 10 μM.
Ethyl gallate	*Phyllodium pulchellum*	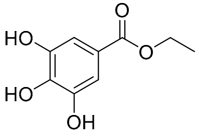	Ref [47]:<br>Vero: IC_{50} > 100 μg/ml;<br>Hela: IC_{50} > 72 μg/ml.
p-Coumaric acid	*Phyllodium pulchellum*	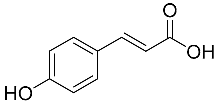	Our test:<br>Hela: IC_{50} > 10 μM;<br>Hep-2: IC_{50} > 10 μM;<br>A549: IC_{50} > 10 μM;<br>RD: IC_{50} > 10 μM.
Caffeic acid ester	*Phyllodium pulchellum*	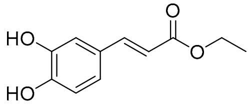	Our test:<br>Hela: IC_{50} > 10 μM;<br>Hep-2: IC_{50} > 10 μM;<br>A549: IC_{50} > 10 μM;<br>RD: IC_{50} > 10 μM.
Daucosterol	*Phyllodium pulchellum*	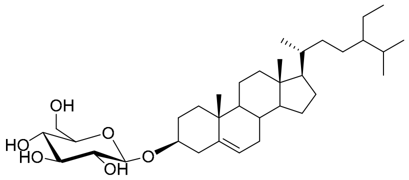	Our test:<br>Hela: IC_{50} > 10 μM;<br>Hep-2: IC_{50} > 10 μM;<br>A549: IC_{50} > 10 μM;<br>RD: IC_{50} > 10 μM.
Loliolide	*Phyllodium pulchellum*	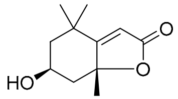	Ref [48]:<br>HL-60: IC_{50} > 100 μM;<br>A-549: IC_{50} > 100 μM;<br>SK-BR3: IC_{50} > 100 μM.

Uridine	*Phyllodium pulchellum*	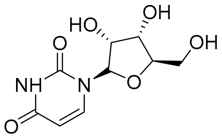	Our test:<br>Hela: $IC_{50} > 10$ µM;<br>Hep-2: $IC_{50} > 10$ µM;<br>A549: $IC_{50} > 10$ µM;<br>RD: $IC_{50} > 10$ µM.
Arbutin	*Phyllodium pulchellum*	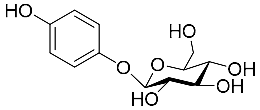	Our test:<br>Hela: $IC_{50} > 10$ µM;<br>Hep-2: $IC_{50} > 10$ µM;<br>A549: $IC_{50} > 10$ µM;<br>RD: $IC_{50} > 10$ µM.

[a] the cytotoxicity data of the 138 compounds derived from coral-derived fungi and plants.

HL-60 = promyelocytic leukemia cell line; K562 = chronic leukemia cell line; A549 = lung carcinoma cell line; CF-1: = spruce budworm cell line; HCT-8 = human colon carcinoma cell line; MDA-MB-435 = human breast cancer cell line; SF-295 = human glioblastoma cell line; Hela = human carcinoma cell line; RD = rhabdomyosarcoma cell line; Hep-2 = epider-moid carcinoma cell line; L5178Y = mouse lymphoma cell line; HepG2 = human hepatoma cell line; HCT-116 = human colon carcinoma cell line; P388 = mouse leukemia cell line; MCF-7 = human breast cancer cell line; NCI-H187 = human small-cell lung cancer cell line; Vero = african green monkey kidney fibroblasts; MDA-MB-231 = human breast cancer cell line; ASPC1 = pancreatic cancer cell line; PC-3 = prostate cancer cell line; SK-MEL-28 = human melanoma cell line; NUGC-3 = human gastric carcinoma cell line; HONE-1 = human nasopharyngeal carcinoma cell line; KB = human oral epidermoid carcinoma cell line; SF-268 = human glioblastoma cell line; NCI-H460 = human small-cell lung cancer cell line; SK-OV-3 = human ovarian cancer cell line; SK-MEL-2 = skin melanoma cell line; XF498 = human CNS tumor cell line; HCT-115 = human colon carcinoma cell line; Hel-299 = human embryonic lung cell line; L1210 = murine leukemia cell line; ASPC = human pancreatic tumor cell line; H460 = human lung cancer cell line; PC3 = prostate cancer cell line; A2780 = human ovarian carcinoma cell line; BC = human breast cancer cell line; L-929 = murine fibroblasts; Neuro-2a = neuronal cell line; Huh-7: human hepatoma cell line; BEL-7402 = human hepatocellular carcinoma cell line; KBv200 = human multidrug-resistant cell line; PC12 = rat pheochromocytoma cell line; HSC-2 = human oral tumor cell line; BGC-823 = stomach cancer cell line; HT-29 = human colon carcinoma cell line; Caco-2 = human colon carcinoma cell line; SW480 = human colon carcinoma cell line; Hep-3B = human hepatoma cell line.

References

1. Qi, J., Shao, C.L., Li, Z.Y., Gan, L.S., Fu, X.M., Bian, W.T., Zhao, H.Y., Wang, C.Y. Isocoumarin derivatives and benzofurans from a sponge-derived *penicillium* sp. fungus. *J. Nat. Prod.* **2013**, *76*, 571–579.
2. Findlay, J.A., Li, G.Q., Miller, J.D., Womiloju, T.O. Insect toxins from *spruce endophytes*. *Can. J. chem.* **2003**, *81*, 284–292.
3. Gallo, M.B.C., Chagas, F.O., Almeida, M.O., Macedo, C.C., Cavalcanti, B.C., Barros, F.W.A., Moraes, M.O.D., Costa-Lotufo, L.V., Pessoa, C., Bastos, J.K., Pupo, M.T. *Endophytic* fungi found in association with *smallanthus sonchifolius* (asteraceae) as resourceful producers of cytotoxic bioactive natural products. *J. Basic Microb.* **2009**, *49*, 142–151.
4. Damour, H., Okoye, F.B.C., Proksch, P., Hakiki, A., Mosaddak, M., Hegazy, M.F., Debbab, A. Pretrichodermamide A and Nafuredin from *Trichoderma* sp, an endophyte of *Cola Nitida*. *J. Mater. Environ. Sci.* **2015**, *6*, 779–783.

5. Wu, G.W., Yu, G.H., Kurtán, T., Mándi, A., Peng, J.X., Mo, X.M., Liu, M., Li, H., Sun, X.H., Li, J., Zhu, T.J., Gu, Q.Q., Li, D.H. Versixanthones A–F, cytotoxic xanthone-chromanone dimers from the marine-derived fungus *Aspergillus versicolor* HDN1009. *J. Nat. Prod*. **2015**, *78*, 2691−2698.
6. Proksa, B., Uhrín, D., Adamcová, J., Fuska, J. Vermixocins A and B, two novel metabolites from *Penicillium vermiculatum*. *J. Antibiot.* **1992**, *45*, 1268−1272.
7. Isaka, M., Chinthanom, P., Veeranondha, S., Supothina, S., Luangsa-ard, J. J. Novel cyclopropyl diketones and 14-membered macrolides from the soil fungus *Hamigera avellanea* BCC 17816. *Tetrahedron* **2008**, *64*, 11028−11033.
8. Wang, M., Chen, Y.C., L. H.H., Sun, Z.H., Tan, G.H., Yan, H.J., Zhang, W.M., Guo, X.L., Study on secondary metabolites of endophytic fungus *bipolaris sorokiniana* from *pogostemon cablin* and their antitumor activities. *J. Guangdong Pharm. Univ.* **2015**, *31*, 576−580.
9. Silva, I.P.D., Brissow, E., Kellner Filho, L.C., Senabio, J., Siqueira, K.A.D., Filho, S.V., Damasceno, J.L., Mendes, S.A., Tavares, D.C., Magalhães, L.G., Junior, P.A.S., Januário,A.H., Soares, M.A. Bioactive compounds of *Aspergillus terreus*-F7, an endophytic fungus from *Hyptis suaveolens* (L.) Poit. *World J. Microb. Biot.* **2017**, *33*, 62.
10. An, X., Pei, Y.H., Chen, S.F., Li, S.G., Hu, X.L., Chen, G., Lin, B., Wang, H.F. Three New Butenolides from the Fungus *Aspergillus* sp. CBS-P-2. *Molecules* **2016**, *21*, 1361.
11. Wang, L., Qin, D., Zhang, K., Huang, Q., Liu, S., Han, M.J., Dong, J.Y. Metabolites from the co-culture of nigranoic acid and *Umbelopsis dimorpha* SWUKD3. 1410, an endophytic fungus from *Kadsura angustifolia*. *Nat. Prod. Res.* **2017**, *31*, 1414−1421.
12. Zhuravleva, O.I., Afiyatullov, S.S., Vishchuk, O.S., Denisenko, V.A., Slinkina, N.N., Smetanina, O.F. Decumbenone C, a new cytotoxic decaline derivative from the marine fungus *Aspergillus sulphureus* KMM 4640. *Arch. Pharm. Res.* **2012**, *35*, 1757−1762.
13. Sun, D.W., Cao, F., Liu, M., Guan, F.F., Wang, C.Y. New fatty acid from a gorgonian-derived *Xylaria* sp. fungus. *Chem. Nat. Compd*. **2017**, *53*, 227−230.
14. Lee, T.H., Chiou, J.L., Lee, C.K., Kuo, Y.H. Separation and determination of chemical constituents in the roots of *Rhus javanica* L. var. *roxburghiana*. *J. Chin. Chem. Soc.* **2005**, *52*, 833−841.
15. Lai, D.W., Brötz-Oesterhelt, H., Müller, W.E., Wray, V., Proksch, P. Bioactive polyketides and alkaloids from *Penicillium citrinum*, a fungal endophyte isolated from *Ocimum tenuiflorum*. *Fitoterapia* **2013**, *91*, 100−106.
16. Khamthong, N., Rukachaisirikul, V., Phongpaichit, S., Preedanon, S., Sakayaroj, J. Bioactive polyketides from the sea fan-derived fungus *Penicillium citrinum* PSU-F51. *Tetrahedron* **2012**, *68*, 8245−8250.
17. Zhang, Y.M., Li, H.Y., Hu, C., Sheng, H.F., Zhang, Y., Lin, B.R., Zhou, G.X. Ergosterols from the culture broth of marine *Streptomyces anandii* H41-59. *Mar. drugs* **2016**, *14*, 84.
18. Liu, F., Cai, X.L., Yang, H., Xia, X.K., Guo, Z.Y., Yuan, J., Li, M.F., She, Z.G., Lin, Y.C., The bioactive metabolites of the mangrove endophytic fungus *Talaromyce*s sp. ZH-154 isolated from *Kandelia candel* (L.) Druce. *Planta Med.* **2010**, *76*, 185−189.
19. Kwon, H.C., Zee, S.D., Cho, S.Y., Choi, S.U., Lee, K.R. Cytotoxic ergosterols from *paecilomyces* sp. J300. *Arch. Pharm. Res.* **2002**, *25*, 851−855.
20. Salama, M.M., Kandil, Z.A., Islam, W.T. Cytotoxic compounds from the leaves of *Gaillardia aristata* Pursh. growing in Egypt. *Nat. Prod. Res.* **2012**, *26*, 2057−2062.

21. Hien, N.T.T., Nhiem, N.X., Yen, D.T.H., Hang, D.T.T., Tai, B.H., Quang, T.H., Anh, H.L.T., Kiem, P.V., Minh, C.V., Kim, E.J., Kim, S.H., Kang, H.K., Kim, Y.H. Chemical constituents of the *Annona glabra* fruit and their cytotoxic activity. *Pharm. Boil.* **2015**,*53*, 1602−1607.
22. Wu, P.L., Lin, F.W., Wu, T.S., Kuoh, C.S., Lee, K.H., Lee, S.J. Cytotoxic and anti-HIV principles from the rhizomes of *Begonia nantoensis*. *Chem. Pharm. Bull.* **2004**, *52*, 345–349.
23. Cai, S.X., Luan, Y.P., Kong, X.L., Zhu, T.J., Gu, Q.Q., Li, D.H. Isolation and photoinduced conversion of 6-epi-stephacidins from *Aspergillus taichungensis*. *Org. Lett.* **2013**, *15*, 2168−2171.
24. Tsukamoto, S., Kato, H., Samizo, M., Nojiri, Y., Onuki, H., Hirota, H., Ohta, T. Notoamides F−K, prenylated indole alkaloids isolated from a marine-derived *Aspergillus* sp. *J. Nat. Prod.* **2008**, *71*, 2064–2067.
25. Kato, H., Yoshida, T., Tokue, T., Nojiri, Y., Hirota, H., Ohta, T., Williams, R.M., Tsukamoto, S. Notoamides A-D: prenylated indole alkaloids isolated from a marine-derived fungus, *Aspergillus* sp. *Angew. Chem.* **2007**, *119*, 2304–2306.
26. Liu, L., Wang, L., Bao, L., Ren, J.W., Bahadur Basnet, B., Liu, R.X,, He, L.W., Han, J.J., Yin, W.B., Liu, H. Versicoamides F–H, prenylated indole alkaloids from *Aspergillus tennesseensis*. *Org. Lett.* **2017**, *19*, 942–945.
27. Chen, M., Shao, C.L., Fu, X.M., Xu, R.F., Zheng, J.J., Zhao, D.L., She, Z.G., Wang, C.Y. Bioactive indole alkaloids and phenyl ether derivatives from a marine-derived *Aspergillus* sp. fungus. *J. Nat. Prod.* **2013**, *76*, 547−553.
28. Li, X.B., Zhou, Y.H., Zhu, R.X., Chang, W.Q., Yuan, H.Q., Gao, W., Zhang, L.L., Zhao, Z.T., Lou, H.X. Identification and biological evaluation of secondary metabolites from the endolichenic fungus *Aspergillus versicolor*. *Chem. Biodiverse.* **2015**, *12*, 575−592.
29. Bunyapaiboonsri, T., Yoiprommarat, S., Intereya, K., Kocharin, K. New diphenyl ethers from the insect pathogenic fungus *Cordyceps* sp. BCC 1861. *Chem. Pharm. Bull.* **2007**, *55*, 304−307.
30. Bünger, J., Westphal, G., Mönnich, A., Hinnendahl, B., Hallier, E., Müller, M. Cytotoxicity of occupationally and environmentally relevant mycotoxins. *Toxicology*, **2004**, *202*, 199−211.
31. Chen, S.T., Wang, J.F., Wang, Z., Lin, X.P., Zhao, B.X., Kaliaperumal, K., Liao, C.J., Tu, Z.C., Li, J.L., Xu, S.H., Liu, Y.H. Structurally diverse secondary metabolites from a deep-sea-derived fungus *Penicillium chrysogenum* SCSIO 41001 and their biological evaluation. *Fitoterapia* **2017**, *117*, 71−78.
32. Rukachaisirikul, V., Satpradit, S., Klaiklay, S., Phongpaichit, S., Borwornwiriyapan, K., Sakayaroj, J. Polyketide anthraquinone, diphenyl ether, and xanthone derivatives from the soil fungus *Penicillium* sp. PSU-RSPG99. *Tetrahedron* **2014**, *70*, 5148−5152.
33. Lin, Z.J., Zhang, G.J., Zhu, T.J., Liu, R., Wei, H.J., Gu, Q.Q. Bioactive cytochalasins from *Aspergillus flavipes*, an endophytic fungus associated with the mangrove plant *Acanthus ilicifolius*. *Helv. Chim. Acta*. **2009**, *92*, 1538−1544.
34. Zhao, D.L., Shao, C.L., Zhang, Q., Wang, K.L., Guan, F.F., Shi, T., Wang, C.Y. Azaphilone and diphenyl ether derivatives from a gorgonian-derived strain of the fungus *Penicillium pinophilum*. *J. Nat. Prod.* **2015**, *78*, 2310−2314.

35. Xia, X.K., Huang, H.R., She, Z.G., Cai, J.W., Lan, L., Zhang, J.Y., Fu, L.W., Vrijmoed, L.L.P., Lin, Y.C. Structural and biological properties of vermistatin and two new vermistatin derivatives isolated from the marine-mangrove endophytic fungus *Guignardia* sp. No. 4382. *Helv. Chim. Acta.* **2007**, *90*, 1925−1931.
36. Chen, M.J., Fu, Y.W., Zhou, Q.Y. Penifupyrone, a new cytotoxic funicone derivative from the endophytic fungus *Penicillium* sp. HSZ-43. *Nat. Prod. Res.* **2014**, *28*, 1544−1548.
37. Xu, J., Kjer, J., Sendker, J., Wray, V., Guan, H.S., Edrada, R., Müller, W.E.G., Lin, W.H., Wu, J., Proksch, P. Cytosporones, coumarins, and an alkaloid from the endophytic fungus *Pestalotiopsis* sp. isolated from the Chinese mangrove plant *Rhizophora mucronata*. *Bioorg. Med. Chem.* **2009**, *17*, 7362−7367.
38. Santos, O.C.S., Soares, A.R., Machado, F.L.S., Romanos, M.T.V., Muricy, G., Giambiagi-deMarval, M., Laport, M.S. Investigation of biotechnological potential of sponge-associated bacteria collected in Brazilian coast. *Lett. Appl. Microbial.* **2015**, *60*, 140−147.
39. Gan, N., Yang, X., Li, T.H., He, P. Studies on constituents of roots and leaves from *Desmodium blandum* and their cytotoxic activity against growth of several tumor cells. *Zhongguo zhongyao zazhi* **2008**, *33*, 2077−2080.
40. Babich, H., Krupka, M.E., Nissim, H.A., Zuckerbraun, H.L. Differential *in vitro* cytotoxicity of (−)-epicatechin gallate (ECG) to cancer and normal cells from the human oral cavity. *Toxicol. In Vitro* **2005**, *19*, 231−242.
41. Tan, Y., Liu, C., Chen, R. Phenolic constituents from stem bark of *Morus wittiorum* and their anti-inflammation and cytotoxicity. *Zhongguo zhongyao zazhi* **2010**, *35*, 2700−2703.
42. Lee, S.B., Cha, K.H., Selenge, D., Solongo, A., Nho, C.W. The chemopreventive effect of taxifolin is exerted through ARE-dependent gene regulation. *Biol. Pharm. Bull.* **2007**, *30*, 1074−1079.
43. Agullo, G., Gamet, L., Besson, C., Demigné, C., Rémésy, C. Quercetin exerts a preferential cytotoxic effect on active dividing colon carcinoma HT29 and Caco-2 cells. *Cancer lett.* **1994**, *87*, 55−63.
44. Alonso-Castro, A.J., Domínguez, F., García-Carrancá, A. Rutin exerts antitumor effects on nude mice bearing SW480 tumor. *Arch. Med. Res.* **2013**, *44*, 346−351.
45. Hsieh, P.W., Chang, F.R., Wu, C.C., Li, C.M., Wu, K.Y., Chen, S.L., Yen, H.F., Wu, Y.C. Longicalycinin A, a new cytotoxic cyclic peptide from *Dianthus superbus* var. *longicalycinus* (Maxim.) Will. *Chem. Pharm. Bull.* **2005**, *53*, 336−338.
46. Babich, H., Sedletcaia, A., Kenigsberg, B. *In vitro* cytotoxicity of protocatechuic acid to cultured human cells from oral tissue: involvement in oxidative stress. *Pharmacol. Toxicol.* **2002**, *91*, 245−253.
47. Kalaivani, T., Rajasekaran, C., Mathew, L. Free radical scavenging, cytotoxic, and hemolytic activities of an active antioxidant compound ethyl gallate from leaves of *Acacia nilotica* (L.) wild. Ex. Delile subsp. *Indica* (Benth.) Brenan. *J. Food Sci.* **2011**, *76*, T144−T149.
48. Pan, X., Matsumoto, M., Nishimoto, Y., Ogihara, E., Zhang, J., Ukiya, M., Tokuda, H., Koike, K., Akihisa, M., Akihisa, T. Cytotoxic and nitric oxide production-inhibitory activities of limonoids and other compounds from the Leaves and Bark of *Melia azedarach*. *Chem. Biodiverse.* **2014**, *11*, 1121−1139.

Table S2 The binding energy of 138 compounds bound with the crystal structure of the ternary complex of topotecan-DNA-Topo I (PDB ID: 1K4T).

Compounds	MOE Dock[a] (kcal/mol)	Ledock[b] (kcal/mol)
S-(−)-5,6,8-Trihydroxy-4-(1′-hydroxyethyl)isocoumarin	−7.7786	−4.16
Sescandelin B	−7.8138	−3.99
Penicimarin E	−8.8656	−4.34
8-Hydroxy-6-methoxy-3-methylisocoumarin	−7.5626	−3.21
Penicimarin B	−8.7150	−4.07
N-(4-Hydroxy-2-methoxyphenyl)acetamide	−7.1517	−3.53
4-Acetoxy-2-methoxyacetanilide	−8.7605	−3.8
S-(−)-2-(2-Hydroxypropanamido)benzamide	−7.4790	−5.01
Nafuredin	−10.5718	−5.05
2-Methoxybenzene-1,4-diol	−5.7260	−2.77
3,4-Dimethoxyphenol	−6.3581	−2.68
Phenyl-acetic acid methyl ester	−6.6832	−2.49
Metyl *p*-hydroxy phenyl acetate	−6.6527	−3.13
3-Methoxy phenol	−5.6058	−2.39
Penicillixanthone A	−11.5816	−6.61
Versixanthone F	−13.9518	−7.26
Bacillosporin C	−11.5710	−5.64
Vermixocin A	−9.0248	−5.34
ω-Hydroxyemodin	−8.9439	−4.97
2-(2,6-Dihydroxybenzoyl)-3-hydroxy-5-hydroxymethylbenzoic acid methyl ester	−9.3932	−5.37
3-Hydroxymethyl-6,8-dihydroxyisocoumarin	−7.1588	−3.72
Di-(2-ethyl)-hexylphthalate	−11.6068	−5.15
Cytosporin L	−8.0071	−5.76
Cytosporin E	−7.5546	−3.93
Cytosporin D	−8.7805	−5.11
N-Acetyltryptamine	−8.5530	−4.33
(11*R*)-2,11,12-Trihydroxy-β-selinene	−6.7288	−4.36
(11*S*)-2,11,13-Trihydroxy-β-selinene	−6.7383	−4.33
Butyrolactone I	−8.9953	−5.31
Butyrolactone II	−8.4991	−5.65
Butyrolactone III	−8.9402	−5.67
Butyrolactone V	−8.7235	−3.74
2,4-Dihydroxy-3,5,6-trimethylbenzoic acid	−7.2234	−3.54
Diorcinol	−8.4427	−4.33
Anodendroic acid	−7.4820	−3.94
Methyl indole-3-carboxylate	−7.1386	−3.82
5-Formylmellein	−7.2639	−3.91

(2*E*,4*E*,6*S*)-6-Hydroxydeca-2,4-dienoic acid	−11.1233	−5.18
7-Chloro-5-hydroxymellein	−7.1996	−3.21
5-Carboxylmellein	−7.2904	−3.64
Sclerotinin A	−8.0502	−3.83
Sclerotinin B	−8.1939	−3.99
Decarboxydihydrocitrinin	−7.5784	−3.83
Dihydrocitrinin	−8.3894	−3.6
(22*E*)-Ergosta-5,7,9(11)22-tetraen-3β-ol	−9.6860	−4.06
Ergosta-5,7,22-triene-3β-ol	−9.8870	−4.77
5α,8α-Epidioxy-ergosta-6,22*E*-dien-3β-ol	−9.8810	−4.96
Altertoxin I	−9.3041	−5.12
6-epi-Stemphytriol	−9.0633	−5.39
7-epi-8-Hydroxyaltertoxin I	−8.4352	−5.7
Stemphyperylenol	−9.3307	−4.99
5α,8α-Epidioxy-ergosta-6,9,22*E*-trien-3β-ol	−9.9299	−4.89
5α,8α-Epidioxy-ergosta-6,22*E*-dien-3β-ol	−10.2242	−4.95
Ergosta-7,22-diene-3β,5α,6β-triol	−11.0112	−5.69
3β,5α-Dihydroxy-6β-methoxyer-gosta-7,22-diene	−10.0029	−5.62
Ergosterol	−9.7855	−4.99
β-Sitosterol glucoside	−12.3521	−7.08
AI-77-C	−11.1595	−6.04
9-Octadecenoic acid-2′,3′-dihydroxy propyl ester	−12.1452	−5.08
3,4-Dihydroxybenzoic acid	−6.0901	−3.37
2,3-Dihydroxybenzoic acid	−5.5446	−3.13
Iindole-3-carboxylic acid	−6.3862	−4.09
6-epi-Stephacidin A	−10.4080	−5.92
Stephacidin A	−9.9812	−6.06
Notoamide B	−9.4178	−5.78
(−)-Versicolamide B	−9.5583	−5.61
Notoamide D	−9.5547	−5.06
Notoamide Q	−10.4351	−6.63
Kipukasin E	−10.4288	−6.02
Kipukasin D	−11.0463	−6.11
Cordyol C	−8.7782	−4.36
Diorcinol F	−8.6403	−4.35
Violaceol II	−8.7515	−4.84
Sterigmatocystin	−9.4854	−4.53
Methyl 6-acetyl-4-methoxy-5,7,8-trihydroxynaphthalene-2-carboxylate	−8.3894	−4.32
3,5-Dichloroasterric acid	−8.6927	−4.89
Penicillither	−8.5524	−4.78

Dihydrogeodin	−8.4534	−4.55
2-(3-Chloro-4-methyl-γ-resorcyloyl)-5-hydroxy-*m*-anisic acid methyl ester	−8.4576	−4.43
Cytochalasin Z16	−9.2543	−4.96
Cytochalasin Z17	−9.3522	−4.87
9,10-Anthracenedione	−9.05	−4.56
Pinophilin D	−10.5462	−6.24
Pinophilin E	−10.3545	−5.90
Pinophilin F	−10.6397	−6.08
Sch 1385568	−10.5149	−5.65
Pinophilin B	−9.6737	−6.07
Sch 725680	−9.5385	−5.56
(−)-Mitorubrin	−10.0119	−5.43
(−)-Mitorubrinol	−10.2443	−5.96
(−)-Mitorubrinic acid	−9.6060	−5.98
Isopenicillide	−8. 9541	−5.54
Purpactin A	−8.7728	−4.93
Vermistatin	−9.8455	−4.61
Methoxyvermistatin	−9.8457	−4.69
4-Demethylvermistatin	−8.9950	−4.57
4-Demethyl-11-methoxyvermistatin	−10.1777	−4.74
Dihydrovermistatin	−10.1114	−4.57
3-*O*-Methylfunicone	−10.5720	−4.8
Penifupyrone	−10.1709	−4.74
Funicone	−10.8324	−4.89
3-*O*-Methyl-5,6-epoxyfunicone	−11.6626	−4.75
Pestalasin A	−8.8730	−3.94
Cyclo(L-Leu-L-Pro)	−8.0535	−3.72
Trichoacorenol	−6.5643	−3.64
Ascotrichic acid	−8.3855	−4.29
Cyclonerotriol	−8.4226	−4.23
5-Hydroxy-*N*,*N*-dimethyltryptamine	−8.0560	−4.49
5-Hydroxy-*N*,*N*-dimethyltryptamine-oxide	−7.9782	−4.71
5-Methoxy-*N*,*N*-dimethyltryptamine	−8.7528	−4.24
5-Methoxy-*N*,*N*-dimethyltryptamine-oxide	−8.8962	−4.61
L-Tryptophan	−7.8726	−5.02
N,*N*-Dimethyl-L-tryptophan	−8.7611	−4.62
2-(Indol-3-yl)ethyl-α-L-rhamnopyra-nosyl-(1→6)-β-D-glucopyranoside	−11.9737	−6.98
2-(Indol-3-yl)ethyl-β-D-glucopyranoside	−9.7652	−5.77
(−)-Epigallocatechin 3-*O*-(*E*)-*p*-coumaroate	−10.9265	−7.15
(−)-Epigallocatechin 3-*O*-(*Z*)-*p*-coumaroate	−10.1798	−7.15

(−)-Gallocatechin	−9.4846	−5.48
(−)-Epigallocatechin	−9.0181	−5.51
(+)-Catechin	−8.2783	−5.24
(−)-Epicatechin	−8.2367	−5.21
(+)-Dihydrokaempferol	−7.9162	−4.76
Dihydroquercetin	−9.4265	−5.26
Quercetin	−9.2606	−5.39
Rutin	−14.1506	−7.7
Quercetin-3-*O*-α-L-rhamnopyranosyl-(1→6)-β-D-galactopyranoside	−14.2123	−8.4
2-*O*-(3,4-Dihydroxybenzoyl)-2,4,6-trihydroxyphenylacetic acid	−8.9730	−5.47
p-Hydroxybenzoic acid	−5.6547	−2.89
Protocatechuic acid	−5.9525	−3.45
Protocaechuic acid methyl ester	−6.6400	−3.36
Protocatechuic acid ethyl ester	−7.2568	−3.65
Ethyl gallate	−7.5169	−3.9
p-Coumaric acid	−6.5956	−3.22
Caffeic acid ester	−7.9648	−3.94
Daucosterol	−12.2041	−6.54
Loliolide	−6.1666	−3.01
Uridine	−7.5324	−4.36
Arbutin	−7.5324	−4.78

[a]MOE Dock: cutoff value at −9.0 kcal/mol. [b]Ledock: cutoff value at −5.0 kcal/mol

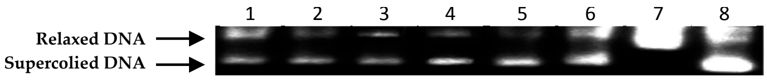

	1	2	3	4	5	6	7	8
pBR322 DNA (0.5 μg)	+	+	+	+	+	+	+	+
TOPO I (1 U)	+	+	+	+	+	+	+	−
CPT (10 μM)	−	−	−	−	−	+	−	−
Compounds (50 μM)	**9**	**4**	**3**	**2**	**1**	−	−	−

Figure S1 DNA Topo I inhibitory activities of (−)-epigallocatechin 3-*O*-(*E*)-*p*-coumaroate (**1**), (−)-epigallocatechin 3-*O*-(*Z*)-*p*-coumaroate (**2**), (−)-epigallocatechin (**3**), quercetin (**4**) and altertoxin I (**9**) at 50 μM. Lanes 1–5: DNA + Topo I + tested compounds; lane 6: DNA + Topo I + CPT; lane 7: DNA + Topo I; lane 8: DNA.

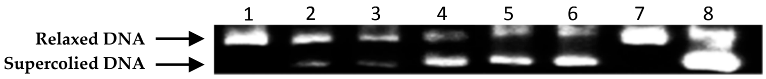

	1	2	3	4	5	6	7	8
pBR322 DNA (0.5 μg)	+	+	+	+	+	+	+	+
TOPO I (1 U)	+	+	+	+	+	+	+	−
CPT (10 μM)	−	−	−	−	−	+	−	−
Compounds (50 μM)	**9**	**4**	**3**	**2**	**1**	−	−	−

Figure S2 DNA Topo I inhibitory activities of (−)-epigallocatechin 3-*O*-(*E*)-*p*-coumaroate (**1**), (−)-epigallocatechin 3-*O*-(*Z*)-*p*-coumaroate (**2**), (−)-epigallocatechin (**3**), quercetin (**4**) and altertoxin I (**9**) at 25 μM. Lanes 1–5: DNA + Topo I + tested compounds; lane 6: DNA + Topo I + CPT; lane 7: DNA + Topo I; lane 8: DNA

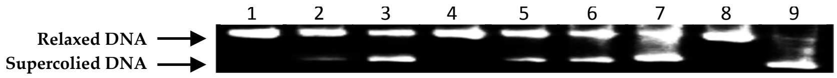

	1	2	3	4	5	6	7	8	9
pBR322 DNA (0.5 μg)	+	+	+	+	+	+	+	+	+
TOPO I (1 U)	+	+	+	+	+	+	+	+	−
CPT (10 μM)	−	−	−	−	−	−	+	−	−
Compound **1** (μM)	−	−	−	1	5	10	−	−	−
Compound **2** (μM)	1	5	10	−	−	−	−	−	−

Figure S3 DNA Topo I inhibitory activities of (−)-epigallocatechin 3-*O*-(*E*)-*p*-coumaroate (**1**) and (−)-epigallocatechin 3-*O*-(*Z*)-*p*-coumaroate (**2**) at 1, 5, and 10 μM. Lanes 1–3: DNA + Topo I + compound **2** at various concentrations; Lanes 4–6: DNA + Topo I + compound **1** at various concentrations; lane 7: DNA + Topo I + CPT; lane 8: DNA + Topo I; lane 9: DNA.

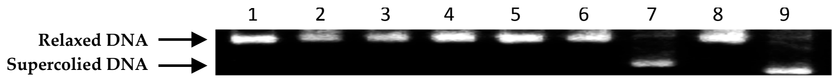

	1	2	3	4	5	6	7	8	9
pBR322 DNA (0.5 μg)	+	+	+	+	+	+	+	+	+
TOPO I (1 U)	+	+	+	+	+	+	+	+	−
CPT (10 μM)	−	−	−	−	−	−	+	−	−
Compound **3** (μM)	−	−	−	1	5	10	−	−	−
Compound **4** (μM)	1	5	10	−	−	−	−	−	−

Figure S4 DNA Topo I inhibitory activities of (−)-epigallocatechin (**3**) and quercetin (**4**) at 1, 5, and 10 μM. Lanes 1–3: DNA + Topo I + compound **4** at various concentrations; Lanes 4–6: DNA + Topo I + compound **3** at various concentrations; lane 7: DNA + Topo I + CPT; lane 8: DNA + Topo I; lane 9: DNA.
